# Insights Into the Somatic Mutation Burden of Hepatoblastomas From Brazilian Patients

**DOI:** 10.3389/fonc.2020.00556

**Published:** 2020-05-05

**Authors:** Talita Ferreira Marques Aguiar, Maria Prates Rivas, Silvia Costa, Mariana Maschietto, Tatiane Rodrigues, Juliana Sobral de Barros, Anne Caroline Barbosa, Renan Valieris, Gustavo R. Fernandes, Debora R. Bertola, Monica Cypriano, Silvia Regina Caminada de Toledo, Angela Major, Israel Tojal, Maria Lúcia de Pinho Apezzato, Dirce Maria Carraro, Carla Rosenberg, Cecilia Maria Lima da Costa, Isabela W. Cunha, Stephen Frederick Sarabia, Dolores-López Terrada, Ana Cristina Victorino Krepischi

**Affiliations:** ^1^International Center for Research, A. C. Camargo Cancer Center, São Paulo, Brazil; ^2^Department of Genetics and Evolutionary Biology, Human Genome and Stem-Cell Research Center, Institute of Biosciences, University of São Paulo, São Paulo, Brazil; ^3^Boldrini Children's Center, Campinas, Brazil; ^4^Department of Biochemistry, Institute of Chemistry, University of São Paulo, São Paulo, Brazil; ^5^Adolescent and Child With Cancer Support Group (GRAACC), Department of Pediatric, Federal University of São Paulo, São Paulo, Brazil; ^6^Department of Pathology and Immunology, Texas Children's Hospital and Baylor College of Medicine, Houston, TX, United States; ^7^Department of Pediatric Oncological Surgery, A. C. Camargo Cancer Center, São Paulo, Brazil; ^8^Department of Pediatric Oncology, A. C. Camargo Cancer Center, São Paulo, Brazil; ^9^Department of Pathology, Rede D'OR-São Luiz, São Paulo, Brazil; ^10^Department of Pathology, A. C. Camargo Cancer Center, São Paulo, Brazil; ^11^Department of Pediatrics, Texas Children's Cancer Center, Houston, TX, United States; ^12^Dan L. Duncan Cancer Center, Baylor College of Medicine, Houston, TX, United States

**Keywords:** hepatoblastoma, *CTNNB1*, *CX3CL1*, *CEP164*, chemokine signaling, cytokine receptor interaction, mutational signature

## Abstract

Hepatoblastoma is a very rare embryonal liver cancer supposed to arise from the impairment of hepatocyte differentiation during embryogenesis. In this study, we investigated by exome sequencing the burden of somatic mutations in a cohort of 10 hepatoblastomas, including a congenital case. Our data disclosed a low mutational background and pointed out to a novel set of candidate genes for hepatoblastoma biology, which were shown to impact gene expression levels. Only three recurrently mutated genes were detected: *CTNNB1* and two novel candidates, *CX3CL1* and *CEP164*. A relevant finding was the identification of a recurrent mutation (A235G) in two hepatoblastomas at the *CX3CL1* gene; evaluation of RNA and protein expression revealed upregulation of *CX3CL1* in tumors. The analysis was replicated in two independents cohorts, substantiating that an activation of the *CX3CL1/CX3CR1* pathway occurs in hepatoblastomas. In inflammatory regions of hepatoblastomas, CX3CL1/CX3CR1 were not detected in the infiltrated lymphocytes, in which they should be expressed in normal conditions, whereas necrotic regions exhibited negative labeling in tumor cells, but strongly positive infiltrated lymphocytes. Altogether, these data suggested that *CX3CL1/CX3CR1* upregulation may be a common feature of hepatoblastomas, potentially related to chemotherapy response and progression. In addition, three mutational signatures were identified in hepatoblastomas, two of them with predominance of either the COSMIC signatures 1 and 6, found in all cancer types, or the COSMIC signature 29, mostly related to tobacco chewing habit; a third novel mutational signature presented an unspecific pattern with an increase of C>A mutations. Overall, we present here novel candidate genes for hepatoblastoma, with evidence that *CX3CL1/CX3CR1* chemokine signaling pathway is likely involved with progression, besides reporting specific mutational signatures.

## Introduction

Hepatoblastoma (HB) is the most common malignant liver tumor in the pediatric population (1), supposedly derived from hepatocyte precursors (2). Although rare, there is a trend toward an increasing prevalence of HBs over the last years (3). The cause of this rising in incidence is still unknown, but a possible explanation would be the increasing survival of premature children with low birth weight, a factor associated with increased risk of HB (4). According to the Children's Hepatic Tumors International Collaboration (CHIC) surveys (5, 6), ~20–30% of children with HBs have resectable tumors at the time of diagnosis. In the last years, almost all children with HB were treated with neoadjuvant and postadjuvant chemotherapy, which raised the overall 5-year survival to ~80% (7, 8). In Brazil, the estimated global survival rate is ~70%, according to INCA (9). Patients who do not respond to standard treatment have very low survival rate (10–13). Few cases in adults have also been described (14–16), and prognosis is most unfavorable. The CHIC has proposed a novel risk stratification system on the basis of prognostic features present at diagnosis (5, 6). Five backbone groups were defined according to clinical prognostic factors—age, α-fetoprotein level (≤ 100 ng/mL), PRETEXT group (I, II, III, or IV), and metastasis at diagnosis.

Hepatoblastoma genomes are relatively stable, with few cytogenetic alterations, mostly gains of chromosomes 2, 8, or 20 (17–19). The major driver mutations in HB tumorigenesis are mainly activators of the WNT pathway, with recurrent mutations in *CTNNB1* (20–23). Few other molecular mechanisms engaged in HB tumorigenesis include overexpression of *IGF2* (24) and its transcriptional activator *PLAG1* (25) and downregulation of *RASSF1A* by promoter hypermethylation (26). This relative paucity of molecular biomarkers in HBs poses a challenge to proper stratification and adjustment of the therapeutic regimen, and molecular subclassification including gene signatures that could be used to stratify patients with HB was reported in the last years (2, 20, 27).

Exome sequencing has broadened the understanding of the HB mutational profile (20, 28–31). The commonalities disclosed by these studies, besides *CTNNB1* mutations, were the low number of detectable somatic mutations, and pathogenic variants in genes from the WNT pathway, such as *CAPRIN2* (28). Other mutations were involved with the ubiquitin ligase complex (*SPOP, KLHL22, TRPC4AP*, and *RNF169*) (28) and with the transcription factor *NFE2L2*, impairing the activity of the KEAP1/CUL3 complex for proteasomal degradation (20, 29). Clinically, overexpression of *NQO1*, a target gene of *NFE2L2*, was significantly associated with poor outcome, metastasis, vascular invasion, and the adverse prognostic C2 gene signature (27). Other two exome analysis were based on syndromic patients who developed HB, including a boy with Simpson–Golabi–Behmel syndrome carrier of a germline *GPC3* loss-of-function mutation (30), and a girl presenting severe macrocephaly, facial dysmorphisms, and developmental delay, in which a novel *de novo* germline nonsense mutation was detected in the *WTX* (31). In a recent study (32), 16 HBs were included in a Pan-Cancer cohort of pediatric tumors, with the identification of *CTNNB1* and *TERT*, genes already known to be frequently mutated in this type of tumor.

We describe here the exome findings and mutational signatures of 10 HBs, disclosing somatic mutations relevant as well as revealing a potential new biological mechanism, corroborated by expression and protein analyses. In addition, germline mutations were investigated in a rare HB presented as congenital disease.

## Materials and Methods

### Patients

This study was approved by Research Ethics Committee—A. C. Camargo Cancer Center, (number 1987/14). Participants and/or persons responsible signed an informed consent form.

All methods were performed in accordance with the relevant guidelines and regulations.

Fresh-frozen tumor and matched non-tumoral liver tissues and blood samples were retrieved from 10 HB patients of the A. C. Camargo Cancer Center Biobank (10 HB samples = exome cohort, five matched non-tumoral liver tissues, and five matched blood samples). A validation cohort was used for targeted sequencing, and RNA expression studies, comprising 12 additional HB cases (11 from GRAACC—Adolescent and Child with Cancer Support Group—and one from A. C. Camargo Cancer Center; clinical features of this second HB cohort are described in Supplementary Table 1). All patients received presurgery chemotherapy according to both SIOPEL (http://www.siopel.org/) and COG (https://www.childrensoncologygroup.org/) protocols. This work was approved by the A. C. Camargo Cancer Center and GRAACC ethics committee; samples were collected after signed informed consent was obtained from parents. Patients were followed by clinical examination, imaging tests, and α-fetoprotein dosage.

In addition to the Brazilian HBs cohorts, a validation set of 16 additional HBs was tested (Supplementary Table 1; TCH samples). All these samples were deidentified specimens selected from the Texas Children's Hospital Department of Pathology archives (Molecular Oncology Laboratory), after institutional review board approval (Baylor College of Medicine Institutional Review Board).

### DNA and RNA Isolation

DNA and RNA were extracted from liver and blood samples following the technical and ethical procedures of the A. C. Camargo Tumor Bank (33, 34), using QIASymphony DNA Mini kit (QIAGEN) and RNeasy Mini Kit (QIAGEN). From tissue embedded in paraffin, direct cut (10 μg) and phenol–chloroform extraction were applied. Purity and integrity of DNA samples were checked by electrophoresis in 0.8% agarose gels and spectrophotometry (NanoDrop; Thermo Scientific, Waltham, MA), and RNA samples were evaluated by microfluidics-based electrophoresis (Bioanalyzer; Agilent Technologies, Santa Clara, CA); only high-quality RNA samples (RIN >7.0) were used for gene expression analysis.

### Exome Sequencing Analysis

Exome data (sequences from protein coding genes of the human genome) were obtained from genomic libraries of 10 HBs and matched non-tumoral samples, enriched using the Sureselect 244K V3 (Agilent Technologies; *n* = 11), OneSeq Constitutional Research Panel (Agilent Technologies; *n* = 5), and QXT SureselectV6 (Agilent Technologies; *n* = 4). Enriched libraries were sequenced on the Illumina HiSeq2500 platform using a 150-bp paired-end protocol to produce a median coverage depth on target of at least 50× per sample. Reads were mapped to their location in the human genome hg19/Grch37 build using the Burrows-Wheeler Aligner package version 0.7.17. Local realignment of the mapped reads around potential insertion/deletion (indel) sites was carried out with the Genome Analysis Tool Kit (GATK) version 3.8. Duplicated reads were marked using Picard version 2.18, reducing false-positive Single Nucleotide Polymorphism (SNP) calls. Additional BAM file manipulations were performed with Samtools 1.7. Base quality (Phred scale) scores were recalibrated using GATK's covariance recalibration. Somatic SNPs and indel variants were called using the GATK Mutect2 for each sample. A total of 53.43 gigabases of sequence data were aligned at high quality (95% of aligned reads), with a mean of 4.45 Gb per sample. More than 95% of the sequenced bases presented Phred score >20. An average coverage depth of 42.6-fold per sample was achieved, with a median of 98% of target regions covered at a minimum of 10× read depth.

Data annotation and filtering variants were run through VarSeq software version 1.5.0 (Golden Helix, Bozeman, MT) using the vcf. files (sequencing data deposited on the public repository of cancer somatic mutations COSMIC under the accession number COSP47849). Variant annotation was performed using different public databases, including population frequency, such as EXAC (http://exac.broadinstitute.org/), gnomAD (Genome Aggregation Database—http://gnomad.broadinstitute.org/), ABRaOM (http://abraom.ib.usp.br/), 1,000 genomes (http://www.1000genomes.org/), and dbSNP version 138 (http://www.ncbi.nlm.nih.gov/projects/SNP/); cancer mutation databases, such as COSMIC version 67 (http://cancer.sanger.ac.uk/cancergenome/projects/cosmic/), ICGC (http://icgc.org/), cBioPortal (https://www.cbioportal.org/), PECAN (https://pecan.stjude.cloud/), and PedcBioPortal (https://pedcbioportal.org/); and clinical sources—Clinvar (https://www.ncbi.nlm.nih.gov/clinvar) and OMIM (https://www.omim.org). Variant filtering was based on quality (Phred score >17), read depth (>10 reads), variant allele frequency (>10%), population frequency (<0.001%), and predicted protein effect [missense, and loss of function (LoF): essential splice site, frameshift, and nonsense variants]. *In silico* prediction of pathogenicity of missense variants was based on six algorithms provided by the database dbNSFP (http://varianttools.sourceforge.net/Annotation/DbNSFP, version 2.4): SIFT (Sorting Intolerant from Tolerant-https://sift.bii.a-star.edu.sg/), Polyphen 2 (Polymorphism Phenotyping v2; http://genetics.bwh.harvard.edu/pph2/), Mutation Taster (http://www.mutationtaster.org/), Mutation Assessor (http://mutationassessor.org/), and FATHMM [Functional Analysis through Hidden Markov Models (V2.3)—http://fathmm.biocompute.org.uk/]. The potential damaging effect was also assessed using the VEP32script software package from Ensembl (https://www.ensembl.org/). Pathogenic/likely pathogenic variants were visually validated as somatic alterations using both Integrated Genomics Viewer (IGV) and Genome Browser (Golden Helix).

Target sequencing was applied for validation of filtered variants; the gene panel was elaborated based on genes disclosed in the current exome analysis (Agilent's SureDesign platform with a total of 18,539 probes and a total size of 498,019 kb). Libraries were prepared from 22 fresh-frozen samples (exome and validation cohorts) using the 244K Agilent SureSelect Target Enrichment (Agilent Technologies) system; the TruSeq v2 chemistry 500 cycles kit was used with 250 pb paired-end-protocol on the Illumina MySeq. The software SureCall (Agilent Technologies) was used for analysis.

### Sanger Sequencing

Prioritized variants from seven candidate genes (from our study and the literature; *CTNNB1, TERT* promoter, *CAPRIN2, CX3CL1, CEP164, AXIN1*, and *DEPDC5*) were validated by Sanger sequencing (sequences upon request) in the HB exome cohort of 10 tumors and investigated in 24 additional samples (12 HBs of the validation group and additional 12 HBs from formalin-fixed paraffin-embedded samples that were contained in a tissue microarray previously made in the institution; the clinical information about the cases included in the tissue microarray is available in Supplementary Table 2). Fourteen HB cases from the Texas Children's Hospital were screened for the *CX3CL1* variant. Polymerase chain reactions (PCR) were performed using standard conditions [95°C, 5 min (44°C, 30 s; 72°C, 30 s; 72°C, 45 s) × 30 cycle; 72°C, 10 min], and amplicons were sequenced in both directions using an ABI 3730 DNA sequencer (Applied Biosystems, Foster City, CA); sequences were aligned with the respective gene reference sequence using Chromas Lite software (Technelysium, South Brisbane, QLD).

### Gene Expression Analysis

*CX3CL1* and *CX3R1* expression analysis was performed by real-time PCR using exome (*n* = 9) and validation cohorts (*n* = 10) and six liver cancer cell lines (HEPG2, C3A, SNU-387, SNU-423, SNU-449, and SNU-475). RNA-to-cDNA conversion was made using the Applied Biosystems High Capacity RNA to cDNA kit following the manufacturer's protocol. For quantitative PCR, we used TaqMan Universal Master Mix II (Applied Biosystems) with reactions performed on an ABI PRISM 7500 instrument. *18S* was selected as the most stable reference gene among *18S, B2M, GAPDH*, and *ACTA1* genes tested according to geNorm (35). Averages from sample triplicates were compared between groups (tumors and non-tumoral tissue), considering differentially expressed those genes with fold changes ≥|2| through the 2^−Δ*ΔCt*^ relative quantitative method (36), with *p* ≤ 0.05. Mann–Whitney test was applied in the analysis of all tumors and cell lines compared to the control group; paired tumor/normal tissue samples were compared using the Wilcoxon test. All tests were corrected using Bonferroni. Prism 6 software (GraphPad Inc., La Jolla, CA) was used for statistical analyses.

Using the published datasets of gene expression from Sumazin et al. (20) and Cairo et al. (27), an *in silico* gene expression analysis was performed based on genes from the Chemokine signaling pathway [Kyoto Encyclopedia of Genes and Genomes (KEGG) database]. The microarray platform from Sumazin et al. (20) contains 117 of 190 Chemokine signaling pathway genes. Different probes targeting the same genes were averaged followed by hierarchical clustering analysis (Euclidian distance with average linkage).

### Immunohistochemistry

Protein analysis was performed for two genes (*CX3CL1* and *CX3CR1*) using the following antibodies: polyclonal antibody PA5-23062 (*CX3CL1*) and polyclonal antibody PA5-19910 (*CX3CR1*), both from ThermoFisher Scientific Company (Waltham, MA). Reactions were automated in the BenchMark Ultra-VENTANA equipment or manual protocol [Novocastra Novolink kit, Leica Biosystems (Buffalo Grove, IL)]. In total, immunohistochemistry was evaluated in 34 cases: nine HB samples from the exome cohort, 17 additional HBs from the tissue microarray (37), and eight samples from the Texas Children's Hospital cohort, including a lung metastasis sample.

### Mutational Signature Detection

Exome data of HBs and matched non-tumoral tissues were used to detect specific mutational signatures. All somatic single-base substitutions were mapped onto trinucleotide sequences by including the 5′ and 3′ neighboring base contexts to construct a 96 × G matrix of mutations count. Next, we used signeR (38) to estimate the number of mutational processes and their signatures. Cosine similarity score was used to compare the signatures with the Pan-Cancer catalog of 30 signatures in COSMIC database.

## Results

### Characteristics of the Cohort

Clinical features of the cohort of the 10 HBs that were studied by exome sequencing are described in Table 1. None of these patients were diagnosed with conditions known to be associated with increased risk for HBs. The mean age at diagnosis was 24.5 months, excluding one patient who was diagnosed at 17 years (HB28). The cohort includes atypical cases. Patient HB28 was born with mild hepatomegaly and was diagnosed with HB at advanced age (17 years), with local recurrence after 5 years followed by death from disease. Patient HB33, female, had a congenital HB diagnosed at 1 month of age; in addition to congenital HB, the patient was born with unilateral renal agenesis. The patient HB31 also was born with a kidney anomaly (a non-functional left kidney), being diagnosed at 2 years with HB. The fourth atypical case was patient HB46, a syndromic male who was born preterm at 27 weeks (birth weight of 945 g, length of 36 cm, and occipital frontal circumference of 25 cm). Evaluated at the age of 3 years 8 months, he exhibited a manifest global developmental delay, with weight of 13.8 kg (<5th centile); height of 94 cm (5th centile) and occipital frontal circumference of 46 cm (<2nd centile); clinical signs included turribrachycephaly with hypoplastic supraorbital ridges, ocular proptosis, high and narrow palate, dental malocclusion, and right preauricular pit; short neck; surgical scar at the abdomen; deep plantar creases and one café-au-lait spot at the gluteal region; three-dimensional cranial computed tomography scan disclosed pansynostosis, with no signs of cranial hypertension. Germline exome analyses of the patients excluded the presence of pathogenic mutations in known disease genes, including those conditions associated with HB risk. Germline likely pathogenic variants were disclosed only in patients 33 and 46, who will be presented later.

**Table 1:** Clinical features of 10 hepatoblastoma cases investigated by exome sequencing.

**ID/gender/age at diagnosis**	**Histology**	**AFP, ng/mL**	Risk stratification^*^/PRETEXT	**Chemotherapy protocol**	**Transplant**	**Metastasis**	**Relapse**	**Deceased**	**Premature (low birth weight)**	**Other features**	**Type of analysis**
HB15, F, 18 m	Epithelial embryonal	5,668,000	Intermediate/4	NA	Yes	No	No	Yes	No	–	Exome sequencing, mutation screening by Sanger sequencing, RNA expression, and IHC assays
HB16, M, 9 m	Epithelial fetal	824	Intermediate/4	SIOPEL3	No	No	No	No	No	–	Exome sequencing, mutation screening by Sanger sequencing, and IHC assays
HB17, F, 36 m	Epithelial fetal	>400,000	Low/1	SIOPEL3	No	No	No	No	No	–	Exome sequencing, mutation screening by Sanger sequencing, RNA expression, and IHC assays
HB18, M, 9 m	Epithelial and mesenchymal mixed	>200,000	Low/3	SIOPEL3	Yes	No	No	No	No	–	Exome sequencing, mutation screening by Sanger sequencing, RNA expression, and IHC assays
HB28, M, 17 y	Epithelial and mesenchymal mixed	NA	High/4	SIOPEL4	No	No	Yes	Yes	No	Hepatomegaly at birth	Exome sequencing, mutation screening by Sanger sequencing, and RNA expression
HB30, M, 54 m	HB with HCC features	>1,000,000	High/2	SIOPEL4	Yes	Lung	Yes	Yes	No	–	Exome sequencing, mutation screening by Sanger sequencing, RNA expression, and IHC assays
HB31, M, 30 m	Epithelial fetal	742,000	Low/3	NA	No	No	No	No	No	Non-functional kidney	Exome sequencing, mutation screening by Sanger sequencing, RNA expression, and IHC assays
HB32, F, 36 m	Epithelial and mesenchymal mixed	9,328,000	High/4	SIOPEL4	Yes	Lung	No	No	No	–	Exome sequencing, mutation screening by Sanger sequencing, RNA expression, and IHC assays
HB33, F, 1 m	Epithelial embryonal and fetal	28,312,000	Intermediate/2	SIOPEL3	No	No	No	No	No	Congenital HB and unilateral renal agenesis	Exome sequencing, mutation screening by Sanger sequencing, RNA expression, and IHC assays
HB46, M, 28 m	Epithelial and mesenchymal mixed	>200,000	High/4	SIOPEL6	No	Lung	No	No	Yes	Syndromic patient^#^	Exome sequencing, mutation screening by Sanger sequencing, RNA expression, and IHC assays

*F, female; M, male; NA, data not available; AFP, α-fetoprotein; IHC, immunohistochemistry*.

* *According to the CHIC criteria (5, 6)*.

# *Facial dysmorphisms, craniosynostosis, and developmental delay*.

Four cases were classified as high risk according to the CHIC criteria, with three patients presenting pulmonary metastasis at diagnosis; one case (HB30) was classified as subtype HB/hepatocellular carcinoma (HCC) features (2, 39). Three patients died of the disease, including the patient diagnosed at 17 years old, and the patient who developed an HB/HCC features tumor; the third patient (HB15) died of complications of liver transplantation.

### Identification of Somatic Coding Non-synonymous Mutations by Exome Sequencing

The strategy of analysis of the exome sequencing data was designed to identify somatic variants, excluding non-coding and coding synonymous variants. Only LoF and missense somatic mutations, the later predicted to be pathogenic by at least one out of six *in silico* algorithm, were considered in this analysis. A total of 94 somatic non-synonymous mutations were disclosed (92 variants), mapped to 87 genes (Supplementary Table 3), all of them validated either by targeted or Sanger sequencing. Two HBs did not present detectable somatic non-synonymous coding mutations (HB17 and HB28), and the congenital case (HB33) was found to harbor 40% of all identified somatic mutations in this cohort. The mean number of somatic non-synonymous mutations per sample was 9.4. However, excluding the atypical HB33, the median number of somatic non-synonymous variants was 6.2 per tumor; thus, HB33 was also presented separately.

Table 2 presents details of the mutations considered to be pathogenic/likely pathogenic in the set of 10 tumors studied by exome sequencing: six LoF variants (five nonsense, and one frameshift, five of them in a single tumor), and six missense variants (recurrent variants or recurrent genes in different tumors), mutations in the promoter of *TERT* and intragenic *CTNNB1* deletions. Three mutations in *CTNNB1* (c.101G> A: COSM5671; c.101G> T: COSM5670; c.121A> G: COSM5664) and one in *GMPS* (c.1367G>T: COSM1040323) had been already reported in COSMIC. Two tumors (one being the congenital case, and HB32) had the same missense mutation (c.704C>G, A235G) in the *CX3CL1* gene, and *CEP164* different mutations were validated in two cases (HB15 c.1861C>A; HB31 c.3263A>G).

**Table 2:** Description of loss of function and recurrently mutated genes identified in 10 hepatoblastomas by exome sequencing and Sanger sequencing (genomic coordinates according to the GRCh37/hg19 Human Assembly): variant data#, mutation type, effect on protein, and prediction of pathogenicity.

**ID**	**Gene**	**Chr:genomic coordinate (rs)**	**VF (%)**	**RefSeq**	**Variant type**	**AA Change**	**Protein change**	Pathogenicity score^*^
HB15	*CEP164*	11:117258055	14	NM_014956	Missense	c.1861C>A	p.Leu621Met	2/5
HB15	*CTNNB1*	3:41266018_41266241	–	NM_001098210	Deletion	c.13_240del228	p. A5_A80del	5/5
HB31	*CEP164*	11:117267312	17	NM_014956	Missense	c.3263A>G	p.Asp1088Gly	2/5
HB16	*CTNNB1*	3:41266104	21	NM_001098210	Missense	c.101G>A	p.Gly34Glu	3/5
HB28	*TERT*	5:1295250	–	–	–	C250T	Promoter	–
HB30	*TERT*	5:1295250	–	–	–	C250T	Promoter	–
HB33	*CTNNB1*	3:41266104 (rs28931589)	58	NM_001098210	Missense	c.101G>T	p.Gly34Val	3/5
HB46	*CTNNB1*	3:41266104 (rs28931589)	52	NM_001904	Missense	c.101G>A	p.Gly34Glu	4/5
HB18	*CTNNB1*	3:41266124 (rs121913412)	43	NM_001904	Missense	c.121A>G	p.Thr41Ala	3/5
HB32	*CX3CL1*	16:57416454	11	NM_002996	Missense	c.704C>G	p.Ala235Gly	2/5
HB33	*CX3CL1*	16:57416454	40	NM_002996	Missense	c.704C>G	p.Ala235Gly	2/5
HB31	*ACACA*	17:35581924	24	NM_198834	Stop codon	c.3463G>T	p.Glu1155Ter	
HB31	*CTNNB1*	3:41266018_41266627	–	NM_001098210	Deletion	c.14_424del411	p. A5_Y142del	5/5
HB33	*ARVCF*	22:19960467	35	NM_001670	Stop codon	c.2531C>T	p.Trp844^*^	1/5
HB33	*DEPDC5*	22:32215040	40	NM_001242896	Stop codon	c.1699C>T	p.Arg567^*^	1/5
HB33	*MYH7*	14:23893250	17	NM_000257	Stop codon	c.2788G>T	p.Glu930Ter	5/5
HB33	*NOL6*	9:33466636	17	NM_022917	Stop codon	c.2022C>T	p.Trp674^*^	3/5
HB33	*KIAA0319L*	1:35900602	29	NM_024874	Frameshift	c.3042^*^>+T	p.Phe1014X	1/5

*Caption: ID, Identification of the sample in the project; VF, frequency of the variant allele; RD, read depth; AA, amino acid*.

* *The pathogenicity score indicates the number of algorithms that predicted for a given missense variant to be deleterious to the protein function (Polyphen2, SIFT, Mutation Taster, Mutation Assessor Pred, FATHMM Pred)*.

**Figure 1:**
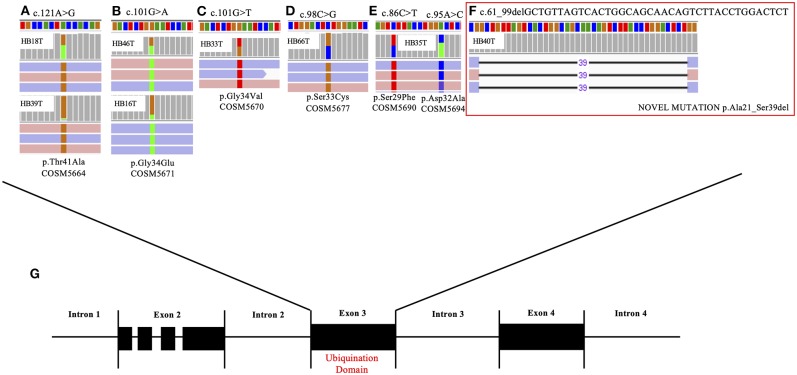
*CTNNB1* somatic mutations detected in eight hepatoblastoma samples. The upper panel presents the six different *CTNNB1* somatic mutations identified by exome sequencing in eight tumors; BAM file images from tumor NGS data show mutations, which were detected in both directions (pink and blue bars correspond to forward and reverse reads, respectively). **(A)** HB18T (variant frequency of 43%) and HB39T (variant frequency of 11%), mutation c.121A> G; **(B)** HB46T (variant frequency of 52%) and HB16T (variant frequency of 21%), mutation c.101G>A. **(C)** HB33T (variant frequency of 58%), mutation c.101G>T. **(D)** HB46T (variant frequency of 50%), mutation c.98C>G. **(E)** HB35T, two mutations: c.86C>T (variant frequency of 49%) and c.95 A>C (variant frequency of 44%); **(F)** HB40T, the novel *CTNNB1* likely pathogenic variant reported in the present study: a 39-bp inframe deletion c.61_99delGCTGTTAGTCACTGGCAGCAACAGTCTTACCTGGACTCT (variant frequency of 21%). **(G)** Detected mutations are all mapped in the exon 3 of the gene, at the ubiquination domain.

Additional 12 HBs were screened for the full set of somatic variants, and only *CTNNB1* mutations were found. In summary, *CTNNB1* alterations were detected in 14 of the 22 tested HBs (64%). Seven *CTNNB1* pathogenic variants were detected in eight samples: six missense mutations (G34E, G34V, T41A, D32A, S29F, and S33C; Figures 1A–E), which had already been reported in HBs (COSMIC), and a novel likely pathogenic variant, a 39-bp inframe deletion (A21_S33del) (Figure 1F, HB40T). All variants map to the *CTNNB1* exon 3 (Figure 1G), at GSK3β phosphorylation sites. Additionally, six tumors presented size variable *CTNNB1* intragenic deletions that were ascertained by Sanger sequencing. Furthermore, previous studies reported that a subset of aggressive HBs carry somatic mutations in the *TERT* promoter region, which could lead to transcriptional upregulation of *TERT* (20, 29, 32); Sanger sequencing disclosed the C250T mutation in two cases of the exome cohort (HB28 and HB30).

Large public databases of cancer genomes, except for COSMIC, do not contain HBs. Therefore, the set of 87 genes harboring somatic mutations was searched in the sequencing data available for HCC samples deposited in the databases ICGC and cBioPortal. As expected, *CTNNB1* had the highest number of mutations in both databases; moreover, in cBioPortal, other 85 genes present mutations at variable frequencies, whereas in ICGC only *TSC2* was also found to be mutated. Searching for our mutated genes in the large pediatric cancer databases PECAN and PedcBioPortal (any tumor) revealed *CTNNB1* as the most commonly mutated gene, followed by *DEPDC5* (several variants were identified in 12 types of pediatric tumors) in PECAN, and *FRMPD1* in PedcBioPortal. Other mutated genes included *ERBB4, EGFR, CEP164*, and *CX3CL1*.

STRING (40) analysis using the 87 mutated genes as seeds and whole genome as background (with all types of evidences with a minimum confidence level of 0.4) revealed an enriched protein–protein interaction network (*p* = 2e-5) involved with some cancers (colorectal, prostate, and endometrial), signaling pathways (thyroid hormone, ErbB, AMPK), adherens junction, choline metabolism in cancer, and proteoglycans in cancer (FDR <0.05, KEGG; Supplementary Figure 1).

Using DNA methylation (DNAm) data recovered from the same group of HB samples (41), *EGFR* and *LMBRD1* genes were hypermethylated in tumors, and *AHRR* was hypomethylated.

To verify if mutations in the 87 genes could impact their expression in HBs and thus have a functional role, expression data were retrieved from two cohorts of HBs and control livers (20, 27). Unsupervised hierarchical clusterization (Euclidian distance with average linkage) based on data from both studies pointed to a disruption of expression of the mutated genes [72 common genes to Cairo et al. (27), and 57 common to Sumazin et al. (20)], because we can observe the grouping of liver tissues relatively separated from HB samples (Supplementary Figure 2).

### Recurrent A235G Somatic Mutation in CX3CL1: A New HB Gene?

**Figure 2:**
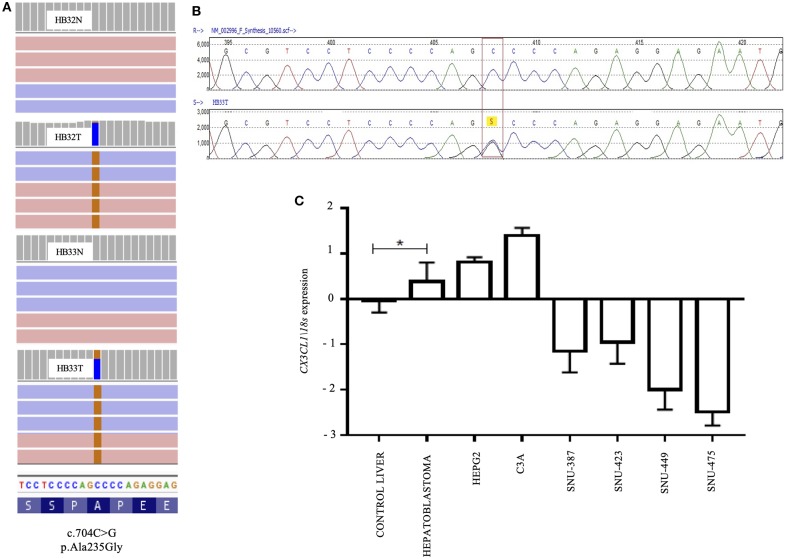
A recurrent A235G somatic mutation detected in the exon 3 of the *CX3CL1* gene and pattern of RNA expression in hepatoblastomas: **(A)** Image obtained from IGV; BAM file images from tumors (HB32T and HB33T) and germinative non-tumoral (HB32N and HB33N) samples showing that the A235G mutations (c.704C>G, p.Ala235Gly) were detected in both directions (pink and blue bars correspond to forward and reverse reads, respectively); HB32T exhibiting a low variant frequency (11%) and HB33T with a variant frequency of 40%. **(B)** Sanger sequencing showing the *CX3CL1* variant in heterozygosity. **(C)** Gene expression pattern of the *CX3CL1* gene in 18 HB samples; HB samples, including the *CX3CL1-*mutated HB32 and HB33 tumors, and the HB cell lines (HEPG2 and C3A) presented upregulation in comparison to control liver samples. The hepatocellular carcinoma cell lines (SNU-387, SNU-423, SNU-449, and SNU-475) were found to be downregulated in relation to control samples and HBs. The statistical test used was Mann–Whitney, *p < 0.05 (Bonferroni correction); endogenous gene: *18s* and the controls are non-tumoral liver tissues. For the analyses, the values in log of relative quantitative were used.

The missense mutation C>G at the position 704 of the exon 3 of *CX3CL1* (NM_002996) was identified in two samples (HB32 and HB33). This mutation, not reported in public databases that document germline variants (gnomAD exomes and genomes, 1K genomes, ABRAOM), leads to substitution of the amino acid alanine by glycine in the codon 235 of the protein, predicted as damaging for protein function by SIFT and Mutation Taster algorithms (Figure 2A). The *CX3CL1* variant was validated by target sequencing in both tumors at heterogeneity (Figure 2B); however, Sanger sequencing detected the mutation only in the tumor sample with the higher variant frequency (HB33, 40%) but did not detect in additional 47 HB samples.

*CX3CL1* expression level was evaluated in 19 HB samples (including the two mutated ones), nine non-tumoral liver samples, two HB cell lines, and four HCC cell lines. Upregulation of *CX3CL1* was detected in the HB group, including *CX3CL1*-mutated tumors and HB cell lines, compared to control liver samples (fold-change >2, *p* < 0.05) (Figure 2C). *CX3CL1* was downregulated in the HCC cell lines compared to control samples and HBs. To investigate if the presence of the *CX3CL1* mutation and/or upregulation of its mRNA could influence the involved pathway, the expression of the *CX3CL1* receptor (*CX3CR1)* was also assessed. Only six tumors presented upregulation of *CX3CR1* mRNA, compared to control (fold-change >2, *p* < 0.05), including a *CX3CL1*-mutated tumor (HB32; Supplementary Figure 3). *CX3CL1* and *CX3CR1* expressions were investigated according to different histological types revealing no association but for HB/HCC sample, which was downregulated for *CX3CL1*, similarly to the HCC cell lines (Supplementary Figure 4).

We performed an *in silico* analysis based on expression data from the studies of Sumazin et al. (20) and Cairo et al. (27). Based on data retrieved from Sumazin et al. (20), *CX3CR1* was downregulated in HBs compared to control liver samples (*p* = 0.0150). Furthermore, the expression values of 190 genes of the Chemokine signaling pathway (KEGG) were submitted to a non-supervised hierarchical clustering analysis based on Euclidian with average linkage, which resulted in grouping of the majority of the HBs (47 of 50 tumors) and discriminated from normal pediatric liver tissues, suggesting that the chemokine signaling pathway is dysregulated in HBs (Supplementary Figure 5). In the data set reported by Cairo et al. (27), *CX3CR1* is listed among the 824 differentially expressed genes between the proposed HB subgroups rC1 and rC2, being upregulated in rC1 (*p* = 0.0001432, FC = 1.7), a group with β-catenin predominantly localized in membrane and cytoplasm.

Data at gene bodies and promoters of *CX3CL1* and *CX3CR1* (41) revealed that DNAm decrease was observed in *CX3CL1* promoter in tumors compared to control liver samples (adjusted *p* = 0.006) and an inverse correlation between gene expression and DNAm level in the *CX3CL1* gene body (Spearman ρ = 0.46, *p* = 0.02), although the latter presented great intertumor heterogeneity (Supplementary Figure 6).

**Figure 3:**
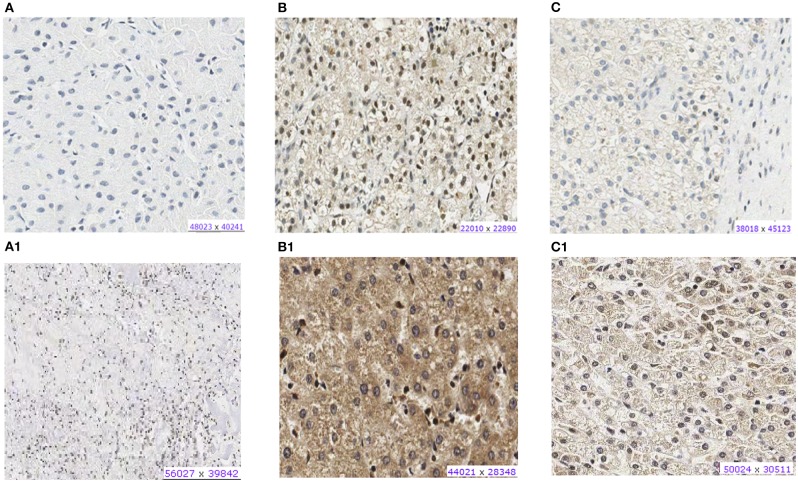
Protein expression of CX3CL1 and CX3CR1 evaluated in hepatoblastoma samples by immunohistochemistry assay. **(A–C)** Show CX3CR1 data, and **(A1–C1)**, CX3CL1 from the same tumor samples. **(A)** HB17, example of negative labeling for CX3CR1 **(A)** and CX3CL1 **(A1)**. **(B)** HB32T, positive for nuclear and cytoplasmic CX3CR1 **(B)** and CX3CL1 **(B1)**. **(C)** HB33T, positive for cytoplasmic CX3CR1 labeling **(C)** and positive for nuclear and cytoplasmic CX3CL1 **(C1)**.

Most HBs (20 of 26) showed CX3CL1 protein expression in the nucleus or cytoplasm (Supplementary Table 4). Both tumors presenting *CX3CL1* mutations presented protein expression: HB32-mutated tumor exhibited weak cytoplasmic labeling and nuclear positivity in more than 50% of cells, whereas HB33-mutated showed strong cytoplasmatic labeling and nuclear positivity in 25% of cells (Figures 3A1–C1); in particular, HB33 exhibited great heterogeneity of histology and protein labeling. Positive labeling of CX3CR1 was detected only in the two CX3CL1-mutated tumors (Figures 3A–C); HB33 showed cytoplasmatic signal, and HB32 had both nuclear and cytoplasmatic labeling. Non-tumoral liver samples did not show any labeling for both proteins.

**Figure 4:**
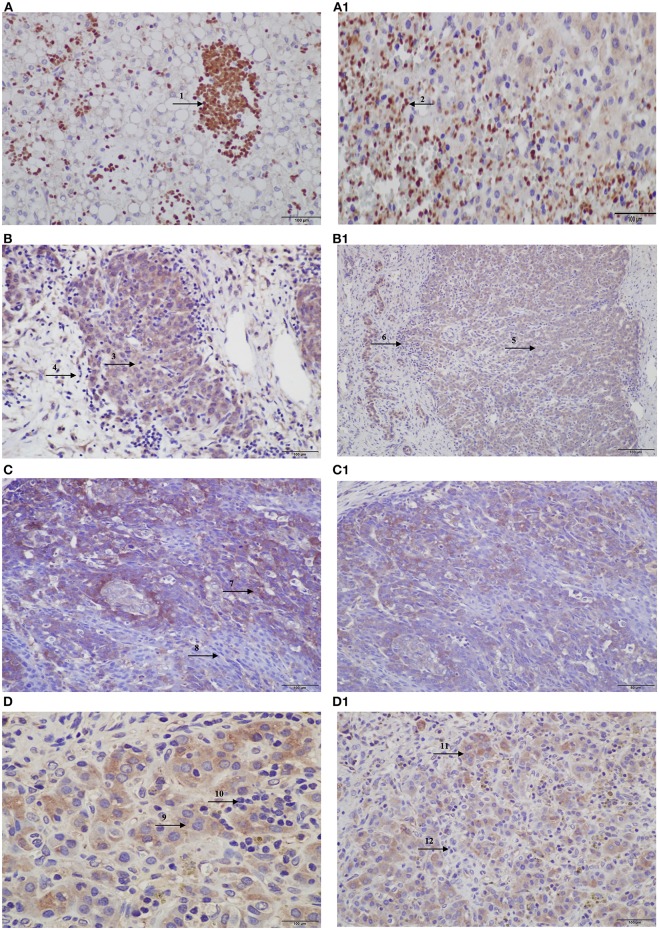
Protein expression of CX3CL1 and CX3CR1 evaluated in hepatoblastomas and hepatoblastoma lung metastasis by immunohistochemistry assay. **(A–D)** Show CX3CL1 data, and **(A1–D1)**, CX3CR1. **(A)** TCH361, CX3CL1 strong positivity of infiltrated lymphocytes (indicated by arrow 1) in necrotic regions of the tumor, and **(A1)**, CX3CR1 strong positivity of infiltrated lymphocytes (indicated by arrow 2) in necrotic regions of the tumor; **(B,B1)** TCH327, positivity in tumor cells (indicated by arrows 3 and 5) and infiltrated lymphocytes negative (indicated by arrows 4 and 6) for both proteins. **(C)** TCH321, positivity in the osteoblast component and strong positivity in the fetal type (indicated by arrow 7); infiltrated lymphocytes are negative (indicated by arrow 8); **(C1)** positivity in tumor cells and lymphocytes negative; **(D,D1)** TCH360, lung metastasis showing positivity in tumor cells (indicated by arrows 9 and 11), and no expression in infiltrated lymphocytes (indicated by arrows 10 and 12), for both proteins.

An independent set of eight HBs and one HB lung metastasis was also evaluated by immunohistochemistry, in a qualitative analysis; the pattern of protein expression was indicative of activation of the *CX3CL1/CX3CR1* pathway, with a predominance of proteins expression in the cytoplasm of tumor cells, similarly to our previous observation (Supplementary Table 5). We also observed that in the inflammatory regions both proteins were not expressed in the infiltrated lymphocytes, in which they should be expressed in physiological conditions, whereas in necrotic regions, the protein staining was negative in tumor cells, but strongly positive in the infiltrated lymphocytes (Figure 4).

### Mutational Signatures of HB

**Figure 5:**
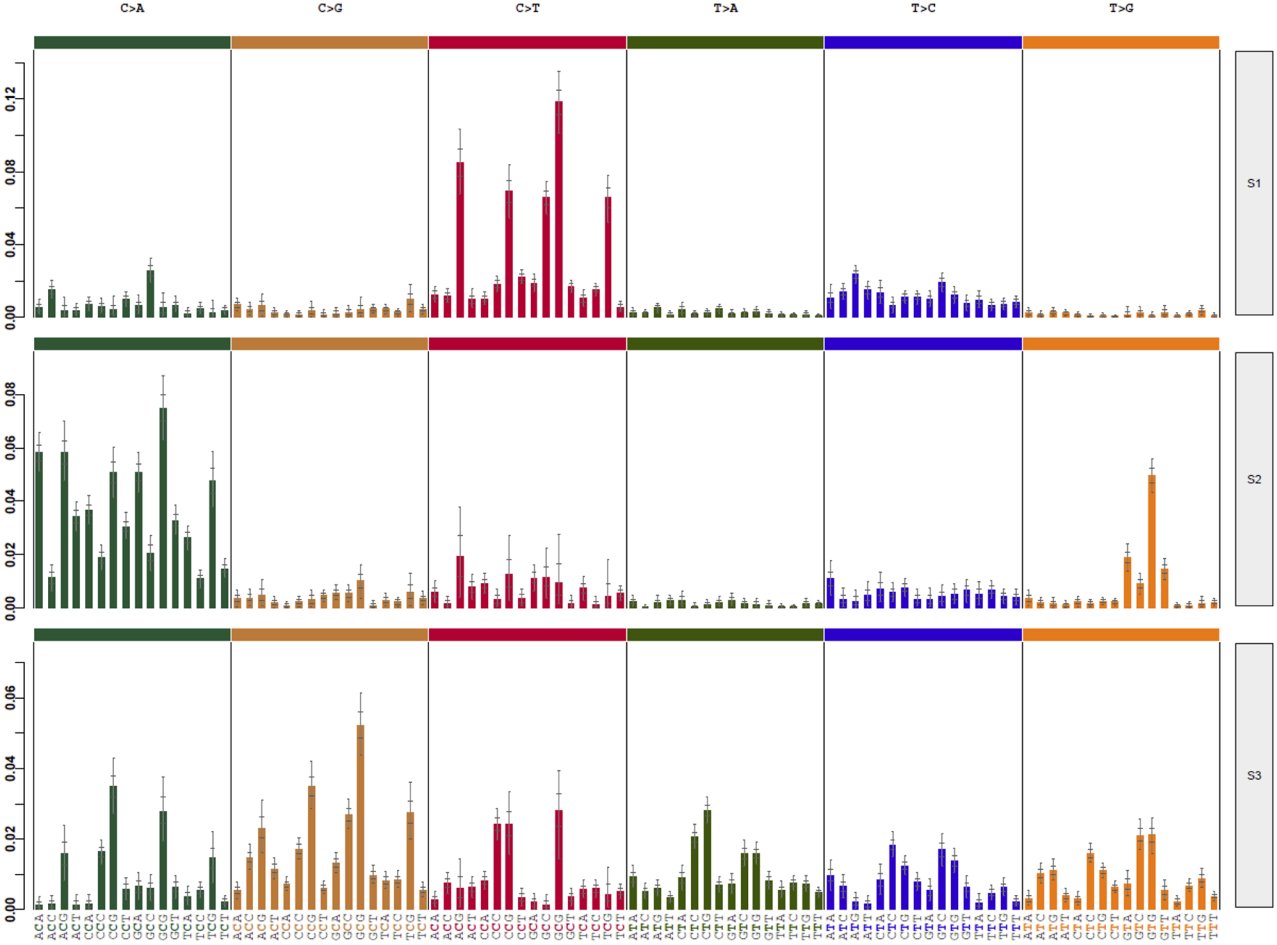
Three different mutational signatures were identified in hepatoblastomas. Exome data of HBs and matched germline tissues were used to detect specific mutational signatures (37). The profile of each signature is displayed using the six substitution subtypes (C>A, C>G, C>T, T>A, T>C, and T>G).

Mutational signatures can reveal properties of underlying mutational processes and are important when assessing signals of selection in cancer. Thus, the presence of single-base substitution signatures was determined for each HB revealing three signatures (HB-S1, S2, and S3, Supplementary Figure 7), two of them presenting great superposition to mutational signatures from COSMIC: HB-S1 group was most similar to COSMIC signatures 1 and 6, and HB-S2 group presented features of the COSMIC signature 29. HB-S3 showed no clear similarity to any known signature, presenting an unspecific pattern with a slight increase of C>A mutations (Figure 5).

### Germline Exome Analysis

#### Congenital HB Case

In addition to the tumor exome, germline exome analysis was performed for this patient and her mother (father was unavailable). We identified 144 rare germline non-synonymous variants in the patient that were absent from her mother (information on frequency and pathogenicity scores of the detected variants are available in Supplementary Table 6a). Twelve germline variants were LoF (*AARSD1, ACSM3, ERI2, CECR2, CRYGA, DNAH7, ETV4, HOXC4, MAMDC4, NEBL, PRSS56*, and *TBXAS1*), standing out a stop gain in *HOXC4*, which was not previously reported in any germline database, including a cohort of Brazilians (ABRAOM), and an indel in the *PRSS56* (ClinVar 31077), both variants already reported in liver cancer samples (ICGC). Additionally, the patient carries six missense variants, which were predicted to be deleterious for protein function using six prediction algorithms, including a variant affecting *BRCA1* and *GOLGA5*, and another one in *FAH* gene not documented in any database (Supplementary Figure 8).

#### Syndromic HB Case

Germline exome analyses were also performed for the syndromic patient HB46 and his parents. Four hundred thirty-five rare non-synonymous variants were identified in the proband (Supplementary Table 6b); 21 of them were LoF (*ALDOB, ANKRD30A, ANKRD36C, ANKRD36C, ARSD, BECN2, BPIFB3, BPIFB4, BSND, CCDC66, CEP89, CRIPAK, FLAD1, GPRC6A, IL17F, MICA, NPC1L1, NT5C1B, PCNX2, RDH5*, and *RNF121*). Among the rare germline variants, we detected a likely pathogenic alteration in the mismatch repair gene *MSH2*, inherited from his mother, and a variant of unknown significance (VUS) in the gene *ABCB11*, inherited from his father (Supplementary Figure 9).

The graphical abstract summarizing the findings can be seen in Supplementary Figure 10.

## Discussion

Our exome results revealed a low mutational background in HBs, corroborating previous works (20, 27, 32), with only three genes presenting recurrent somatic mutations, namely, *CTNNB1, CX3CL1*, and *CEP164*. *CTNNB1* somatic mutations were detected in ~60% of the tumors here studied, including a novel pathogenic variant (A21_S33del). Mutation in *A2ML1* was common to one of the major exome studies of HB (29); however, the role of *A2ML1* somatic mutations remains unclear.

Our data pointed out to a novel set of candidate genes for HB biology with a potential functional role in the HB tumorigenesis as they had an impact in the gene expression levels. Moreover, this gene set was enriched among gene sets from other cancers: EGFR-KRAS-ALK–negative lung adenocarcinoma in never-smokers (*CFTR, CTNNB1, EGFR, ERBB4, MXRA5, TGFBR2*) (42), bladder cancer (*EGFR, ERBB4, FLCN, PIK3R1, TSC2*) (43), and metastatic renal cell carcinoma (*DEPDC5, EGFR, FLCN, PIK3R1, TSC2*) (44), suggesting they could have a broader role in cancer.

*CEP164*, a key element in the DNA damage-activated signaling cascade (45) involved in genome stability, was found to be mutated in two different HBs. *CEP164* is overexpressed in various cancer types, often associated with poor prognosis (46), and a recent study in rhabdomyosarcoma cells suggested a central role of this gene in proliferation in response to cellular stress (47). Remarkably, one of the *CEP164*-mutated HBs exhibited a complex genome, with several copy number alterations and two large LOH regions. Three genes, which we have previously reported as differentially methylated in HBs (41), were mutated in the present cohort, reinforcing a possible role in HB tumorigenesis: *EGFR, LMBRD1*, and *AHRR*. *LMBRD1* encodes a lysosomal membrane protein and is associated with a vitamin B12 metabolism disorder (48), and *AHRR* and *EGFR* are involved in regulation of cell growth and differentiation. Loss-of-function variants were identified in *ACACA, ARVCF, DEPDC5, MYH7, NOL6*, and *KIAA0319L*; nevertheless, all but the *ACACA* variant were detected in the congenital tumor, making difficult to associate these mutations with HB in general.

Although there are some studies with larger cohorts of HBs (20, 28, 29, 32), their sequencing data are not deposited in public databases, hampering further evaluation of the significance of the mutational set from our study. However, several genes were found to be mutated in other pediatric tumors in these databases, such as *CTNNB1, DEPDC5, ERBB4, EGFR, CEP164*, and *CX3CL1*. The only sample classified as subtype HB/HCC features carries alterations in genes found to be mutated in HCCs (*TSC2, HMCN1, UNC80, VPS13B*, and *TERT* promoter), corroborating the histological classification because the mutational load resembles hepatic tumors with more differentiated cells.

The most significant finding in this study was the detection of a recurrent somatic missense mutation in the exon 3 of *CX3CL1*, leading to the substitution of the amino acid alanine by glycine in the protein, and predicted by two algorithms as damaging. This gene, chemokine ligand 1 (C-X3-C motif), encodes a large transmembrane 373-aa multiple-domain protein from the chemokine family, the fractalkine. This protein is present in endothelial cells of diverse tissues, such as brain and kidneys (49), and is related to leukocyte movement, including migration to inflammation sites (50, 51). The cell adhesion and migration functions are promoted through interaction of fractalkine with the chemokine receptor CX3CR1, a transmembrane protein known to provide prosurvival signaling for anti-inflammatory monocytes, but also present in natural killer cells and T cells (52). The amino acid 235 of CX3CL1, in which the mutation occurred, is part of the mucin-like region of the protein, which exerts a key role on its binding to CX3CR1. Under inflammatory response conditions, cleavage of CX3CL1 by metalloproteinases generates a soluble chemokine, which binds to CX3CR1 in nearby cells and can induce adhesion, cell survival, and migration.

The significance of *CX3CL1* mutations in cancer is yet poorly understood, but different mutations in this gene have been reported in other tumor types, predominantly in gastric cancer (COSMIC) and HCCs (TCGA). Gastric tumors exhibit increased *CX3CL1* expression (53), and *CX3CR1* is highly expressed in association with more advanced stages. We showed that *CX3CL1* is upregulated in HBs, a result that was corroborated by immunohistochemistry assays. Only the two *CX3CL1*-mutated tumors presented CX3CR1 protein expression, evidencing an activation of this chemokine signaling pathway. Increased *CX3CL1* expression was also observed in several HBs without detectable *CX3CL1* mutations; this finding suggests that alternative pathways for *CX3CL1* activation exist, and the hypomethylation at the *CX3CL1* promoter disclosed in HBs supports the hypothesis of epigenetic regulation. Considering the observed CX3CL1-CX3CR1 pattern of expression in HBs, we can speculate that the detected missense *CX3CL1* mutation would lead to a gain of function. Xu et al. (54) and Yang et al. (55) published results of other chemokines in liver cancer, with data also indicating an oncogenic role.

Using the published datasets of gene expression from Sumazin et al. (20) and Cairo et al. (27), we found evidence of dysregulation of the chemokine signaling pathway in HBs. In particular, *CX3CR1* exhibited a consistent pattern of downregulation in ours and aforementioned expression studies, but increased expression was observed in those tumors with no strong nuclear β-catenin labeling [subgroup rC1 from Cairo study (27)], suggesting a possible mechanism rather independent of the Wnt signaling pathway activation. Inappropriate expression or regulation of chemokines and their receptors is linked to many diseases, especially those characterized by an excessive cellular infiltrate, such as rheumatoid arthritis and other inflammatory disorders. In recent years, the involvement of chemokines and their receptors in cancer, particularly metastases, has been well-established (56, 57). Chemokines recruit leukocytes, which produce other cytokines, growth factors, and metalloproteinases that increase proliferation and angiogenesis. The metastasis process is facilitated by the regulation of particular chemokine receptors in tumor cells, which allows them to migrate to secondary tissues where the ligands are expressed (58). Our results indicate that the activation of the CX3CL1-CX3CR1 pathway could be related to HB development or progression. In an independent HB group, a contrasting pattern of *CX3CL1* and *CX3CR1* was observed in regions of inflammation in the samples and in areas with necrosis. Around necrotic regions, *CX3CL1* and *CX3CR1* were detected in the infiltrated lymphocytes, indicating a normal immune response; however, in inflammation regions, both proteins were strongly positive in tumor cells and not detected in infiltrated lymphocytes, suggesting a mechanism of regulation of this pathway in favor of HB cells. Studies of chemokines and cancer, especially in liver tumors, suggest that this pattern would be an adaptive mechanism of the tumor cells, “misleading” the immune system and preventing them from acting by fighting the tumor cells. This result further adds to previous studies showing that the activation of the ligand and receptor in chemokines may be involved in tumor invasion (53–55, 59–61).

Recurrent driver mutations in HBs are already well-established, such as mutations in the Wnt pathway genes, mainly *CTNNB1*, and mutations in *NFE2L2* and the promoter of *TERT*. It is hard to establish which of the novel mutations have impact in tumor development due to the variability of the mutational profiles of the tumors, and probably only part of the alterations is actually relevant for HB biology. Particularly, the *CX3CL1* and *CEP164* genes were highlighted because mutations in these genes were recurrent in this cohort, and the role of *CX3CL1* and its receptor was further investigated because two tumors carried the same *CX3CL1* mutation. Considering that *CX3CL1* is not directly related to the Wnt pathway or other HB-related pathways of origin and that most of the tumors included in our study carry one known driver mutation (including one of the *CX3CL1*-mutated tumors), the activation of the CX3CL1/CX3CR1 pathway is more likely linked to chemotherapy response and progression. In fact, at this point, it is not possible to discern whether *CX3CL1* signature would be cause or consequence in HB tumorigenesis, and this provides a starting point for future studies aiming to investigate if the activation of this pathway could be raised by the chemotherapy treatment.

In addition to revealing coding somatic mutations in HBs, exome data were used to search for mutational processes. In general, it was remarkable that the most frequent mutational signatures reported in liver cancer were not observed in these HBs, suggesting distinct mutational mechanisms for HCCs and liver embryonal tumors. Two of the three mutational signatures here observed have superposition mainly with three known signatures from COSMIC (signatures 1, 6, and 29). Signature 29 has been observed mostly in gingiva–buccal oral squamous cell carcinoma developed in individuals with a tobacco chewing habit but was recently reported also for HCC samples; this signature indicates guanine damage that is most likely repaired by transcription-coupled nucleotide excision repair. Among the several chemicals in smokeless tobacco that have found to cause cancer (62), the most harmful carcinogens are nitrosamines, which level is directly related to the risk of cancer and that can be also find in food such as cured meat, smoked fish, and beer. Interestingly, O(6)-methylguanine detected in human cord blood in mothers highly exposed to such products implicates nitrosodimethylamine exposure of the fetus and toxicity from dietary sources of these compounds (63). Maternal dietary exposure to N-nitroso compounds or to their precursors during pregnancy has also been associated with preterm birth (64) and risk of childhood cancer (65). Childhood cancer is most probably the combinatorial result of both genetic and environmental factors, and these networks between fetal exposure to environmental carcinogens such as nitrosamines from tobacco and/or dietary sources, preterm birth, and increased risk of childhood cancer may be an underlying cause for at least a subset of HBs. Finally, a subset of tumors, including two patients who died of the disease, exhibited a mutational pattern with no clear similarity to any known signature.

As a final point, we analyzed in detail the germline exomes of two patients. One of them was the patient with a congenital HB and unilateral renal agenesis, who developed a tumor exhibiting a heterogeneous histology (HB33). This tumor carried the highest number of somatic mutations herein detected, including *CX3CL1* and *CTNNB1* mutations, and its chromosome copy number profile was complex compared to the HB group (data not shown). In addition to very rare germline variants in genes related to liver function, such as *HOXC4, PRSS56*, and *CYP1A1*, the patient carried two variants strongly predicted to be deleterious affecting *BRCA1* and *FAH*, both genes associated with cancer predisposition (66). In particular, the *FAH* gene encodes a fumarylacetoacetate hydrolase enzyme that is mainly abundant in liver and kidneys (67), and germline mutations were already reported to increase the risk of HCC (68), although only in a recessive mode of inheritance. In the second patient (HB46), a syndromic male with craniosynostosis and dysmorphic signs, another *CYP1A1* variant mapping in the same exon that the one observed in the previous patient was detected. In addition, two relevant germline alterations were disclosed: a likely pathogenic missense variant in *MSH2*, which is involved in DNA mismatch repair, and a VUS affecting *ABCB11*, associated with an autosomal recessive disorder (progressive familial intrahepatic cholestasis). *MSH2* heterozygous mutations can result in hereditary non-polyposis colorectal cancer (69), Muir–Torre syndrome, and mismatch repair cancer syndrome (70, 71). *ABCB11* mutations also confer increased risk of developing HCC (72–77), but only in a recessive mode of inheritance, such as the *FAH* gene. Interestingly, emerging evidences suggest that individuals harboring germline variants in heterozygosity in autosomal recessive cancer predisposition genes may also be at increased cancer risk (78–83).

Recent studies have corroborated previous observations of increased risk of pediatric cancer in a child with birth defects and/or skin tags unrelated to chromosomal abnormalities or known genetic syndromes (75–77). There are also descriptions of specific associations, such as increased risk of lymphoma in children with congenital heart defects, especially correlated with complex conditions, suggesting a shared origin in the development of the two conditions (78). These findings strongly suggest that pediatric/embryonal tumors and congenital anomalies share common etiologic factors underlying their development, and this is a relevant and ongoing discussion in the literature. Particularly, there is a relevant association between craniosynostosis and renal/genital anomalies with HB development (76–80), suggesting a yet unknown common molecular mechanism. In our cohort, five patients exhibited congenital renal anomalies, and the syndromic patient had craniosynostosis.

Several lines of evidence indicate that childhood and adult cancers are distinct entities. Despite intensive efforts, relevant genetic factors remain difficult to be captured in rare cancers as embryonal tumors such as HB. In summary, in this study, we provide evidences that the activation of the *CX3CL1/CX3CR1* chemokine signaling pathway can be involved in HB progression or response to chemotherapy. We also present the first assessment of mutation signatures in HBs identifying a novel signature specific to a subset of these tumors.

## Data Availability Statement

The data in this article can be found in the COSMIC database (https://cancer.sanger.ac.uk/cosmic) using the accession number COSP47849.

## Ethics Statement

This study was approved by Research Ethics Committee—A. C. Camargo Cancer Center (number 1987/14). Written informed consent to participate in this study was provided by the participants' legal guardian/next of kin. Written informed consent was obtained from the individual(s), and minor(s)' legal guardian/next of kin, for the publication of any potentially identifiable images or data included in this article.

## Author Contributions

TA, TR, CR, and AK: conception and design. TA, MR, SC, TR, JS, AB, AM, DB, SS, MC, SRC, ML, DC, CR, CL, IC, D-LT, and AK: collection and assembly of data. TA, MR, SC, TR, JS, AB, MM, RV, GF, DB, SS, D-LT, IT, DC, CR, CL, IC, and AK: data analysis and interpretation. TA, CR, and AK: manuscript writing. All authors: final approval of manuscript.

## Conflict of Interest

The authors declare that the research was conducted in the absence of any commercial or financial relationships that could be construed as a potential conflict of interest.
