# Supplementary material for: Insights Into the Somatic Mutation Burden of Hepatoblastomas From Brazilian Patients
Source: Front Oncol. 2020 May 5;10:556. doi: 10.3389/fonc.2020.00556 (PMC7214543; doi:10.3389/fonc.2020.00556)
Supplement: Supplementary file 1 [file Data_Sheet_1.PDF]

SUPPLEMENTARY DATA

INSIGHTS INTO THE SOMATIC MUTATION BURDEN OF HEPATOBLASTOMAS FROM BRAZILIAN PATIENTS

Talita Ferreira Marques Aguiar[1,2], Maria Prates Rivas[2], Silvia Costa[2] , Mariana Maschietto[3] , Tatiane Rodrigues[2], Juliana Sobral de Barros[2], Anne Caroline Barbosa[2], Renan Valieris[1], Gustavo R. Fernandes[4], Debora R. Bertola[2], Monica Cypriano[5] , Silvia Regina Caminada de Toledo[5], Angela Major[6], Israel Tojal[1] , Maria Lúcia de Pinho Apezzato[7] , Dirce Maria Carraro[1], Carla Rosenberg[2], Cecilia Maria Lima da Costa[8],Isabela W. Cunha[9,10], Stephen Frederick Sarabia[6], Dolores-López Terrada[6,11,12], Ana Cristina Victorino Krepischi[2,] *

1. International Center for Research, A. C. Camargo Cancer Center, São Paulo, BR
2. Human Genome and Stem-Cell Research Center, Department of Genetics and Evolutionary Biology, Institute of Biosciences, University of São Paulo, São Paulo, BR
3. Boldrini Children's Center, Campinas, BR
4. Department of Biochemistry, Institute of Chemistry, University of São Paulo, São Paulo, BR
5. Department of Pediatric, Adolescent and Child with Cancer Support Group (GRAACC), Federal University of São Paulo, São Paulo, BR.
6. Department of Pathology and Immunology, Texas Children’s Hospital and Baylor College of Medicine, Houston, USA
7. Department of Pediatric Oncological Surgery, A. C. Camargo Cancer Center, São Paulo, BR
8. Department of Pediatric Oncology, A. C. Camargo Cancer Center, São Paulo, BR
9. Department of Pathology, Rede D’OR-São Luiz, São Paulo, BR
10. Department of Pathology, A. C. Camargo Cancer Center, São Paulo, BR
11. Texas Children’s Cancer Center, Department of Pediatrics, Houston, USA
12. Dan L. Duncan Cancer Center, Baylor College of Medicine, Houston, USA

***Corresponding author:**
Ana Cristina Victorino Krepischi e-mail: ana.krepischi@ib.usp.br

Keywords: Hepatoblastoma, somatic mutation, *CTNNB1, CX3CL1, CEP164,* Chemokine signaling, Cytokine receptor interaction, mutational signature

A

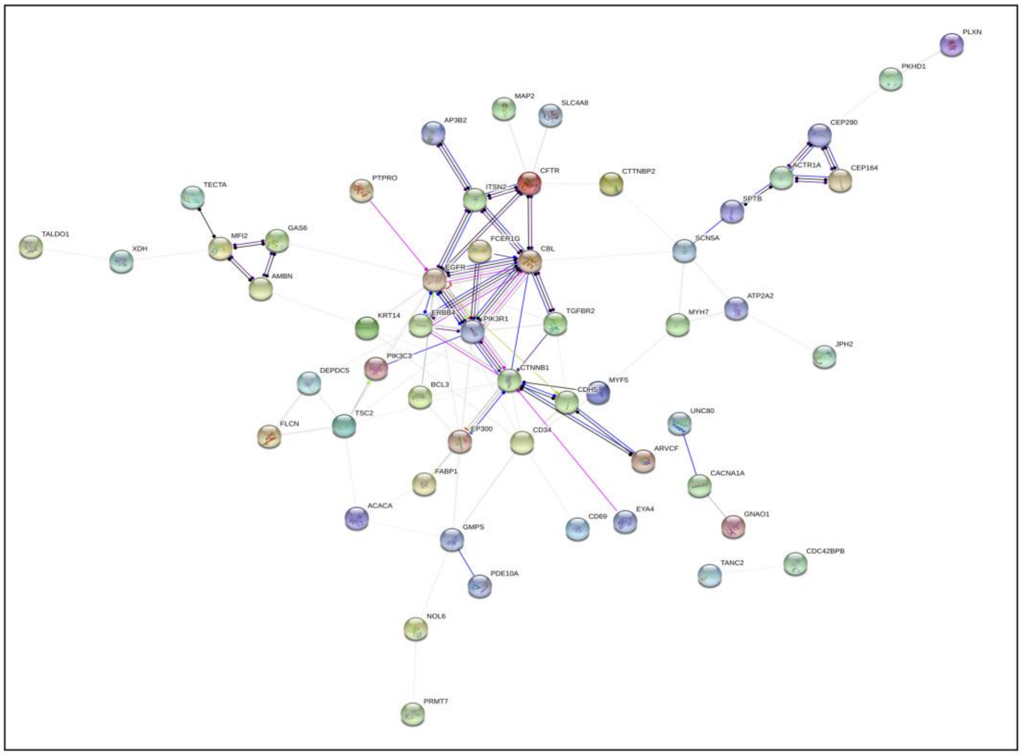

B

**Term description -KEGG**	**Observed gene count**	**False discovery rate**	**Matching proteins**
Adherens junction	4	0.0333	*CTNNB1, EGFR, EP300, TGFBR2*
Thyroid hormone signaling pathway	5	0.0333	*ATP2A2, CTNNB1, EP300, PIK3R1, TSC2*
ErbB signaling pathway	4	0.0346	*CBL, EGFR, ERBB4, PIK3R1*
Colorectal cancer	4	0.0346	*CTNNB1, EGFR, PIK3R1, TGFBR2*
Prostate cancer	4	0.0363	*CTNNB1, EGFR, EP300, PIK3R1*
Choline metabolism in cancer	4	0.0363	*EGFR, PIK3R1, SLC44A1, TSC2*
Proteoglycans in cancer	5	0.0480	*CBL, CTNNB1, EGFR, ERBB4, PIK3R1*
AMPK signaling pathway	4	0.0483	*ACACA, CFTR, PIK3R1, TSC2*
Endometrial cancer	3	0.0483	*CTNNB1, EGFR, PIK3R1*

Supplementary Figure 1: STRING analysis. STRING analysis **(A)** using the 87 mutated genes as seeds and whole genome as background (with all types of evidences with a minimum confidence level of 0.4) revealed an enriched protein-protein interaction (PPI) network (pvalue =2e-5) involved with some cancers (colorectal, prostate and endometrial), signaling pathways (thyroid hormone, ErbB, AMPK), adherens junction, choline metabolism in cancer and proteoglycans in cancer; FDR<0.05 - KEGG – **(B).**

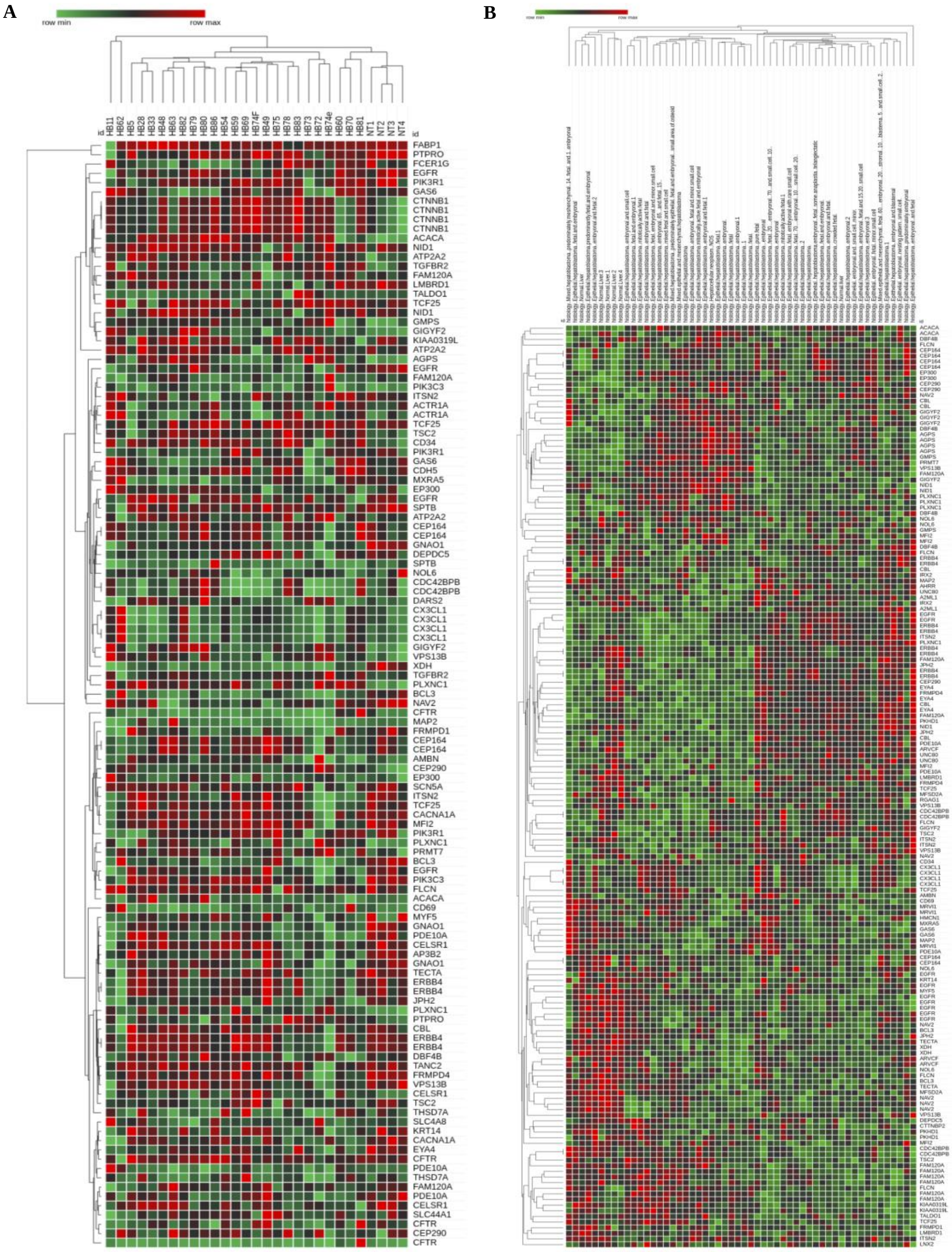

Supplementary Figure 2: Unsupervised hierarchical clusterization based on data from both studies pointed to a disruption of expression of the mutated genes. The microarray platform has 72 common genes to Cairo et al. (27) - shows in **(A)** and 57 common to Sumazin et al. (20) - shows in **(B)**. The mean was calculated for different probes for the same transcripts and used for a non-supervised hierarchical clustering analysis (Euclidian distance with average linkage). In this clustering analysis, the majority of the hepatoblastoma samples were grouped separately from control liver samples (normal pediatric liver tissues).

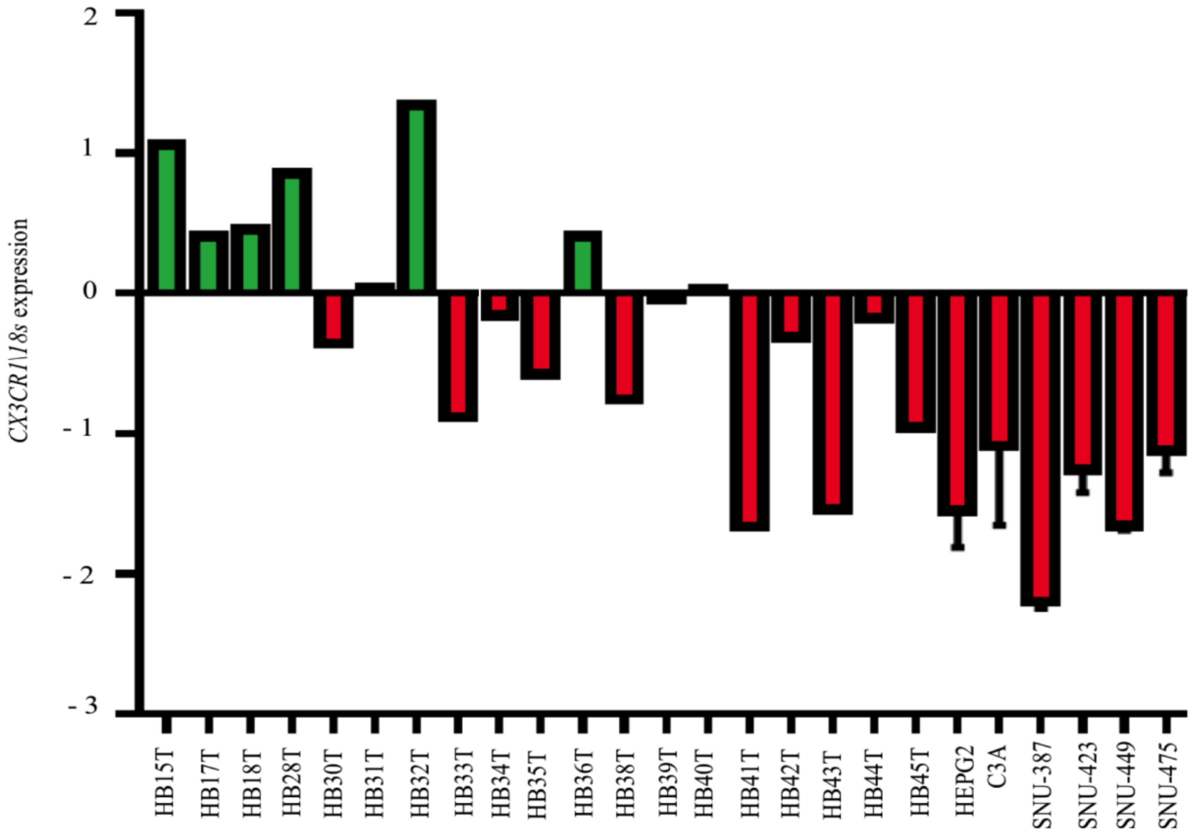

Supplementary Figure 3: Gene expression pattern of the *CX3CR1* gene in 18 HB samples and six liver tumor cell lines. Only six tumors (green bars) presented *CX3CR1* upregulation in comparison to control liver samples. The hepatoblastomas cell lines (HEPG2 and C3A) and the hepatocellular cell lines (SNU-387, SNU-423, SNU-449 and SNU-475) were found to be down-regulated in relation to control samples. The statistical test used was Mann-Whitney, no significance; Endogenous gene: 18s and the controls are non-tumoral liver tissues. For the analyzes the values in log of RQ were used.

A

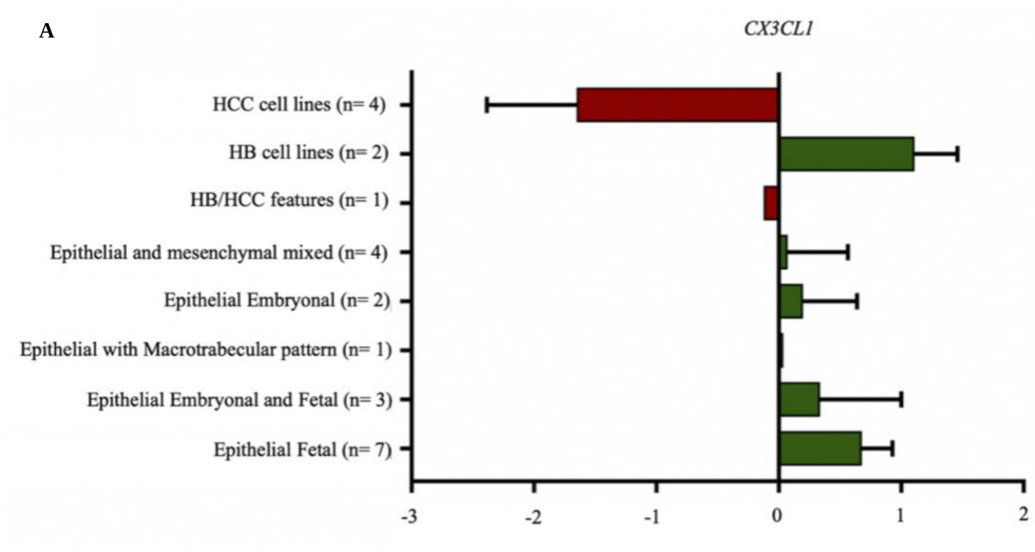

B

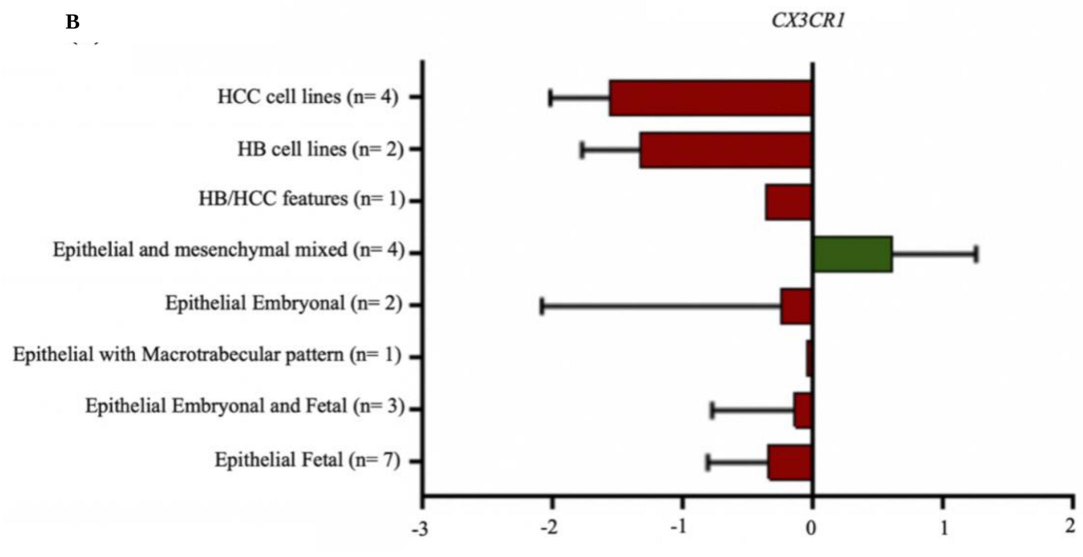

Supplementary Figure 4: Gene expression analysis of CX3CL1/CX3CR1 genes according to different hepatoblastoma histological types. Expression of CX3CL1 **(A)** and CX3CR1 **(B)** in different histological types of hepatoblastomas in comparison to control livers.

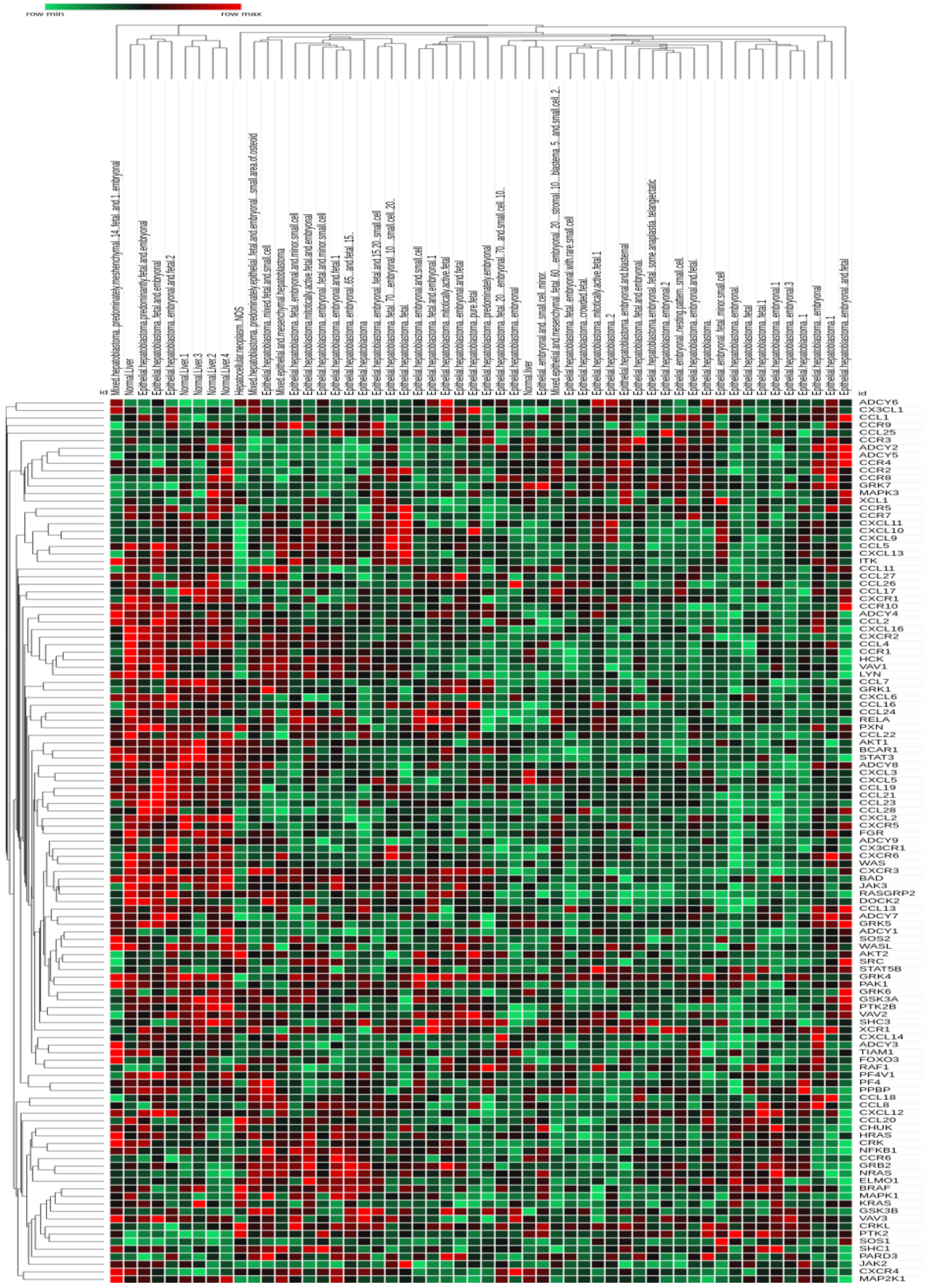

Supplementary Figure 5: Non-supervised hierarchical clustering analysis from the Chemokine signaling pathway in hepatoblastomas. The microarray platform from Sumazin et al. (2017) contains 117 genes of the Chemokine signaling pathway listed in KEGG database. The mean was calculated for different probes for the same transcripts and used for a non-supervised hierarchical clustering analysis (Euclidian distance with average linkage). In this clustering analysis, the majority of the hepatoblastoma samples (47) were grouped separately from control liver samples (normal pediatric liver tissues), suggesting that the Chemokine signaling pathway is dysregulated in tumors.

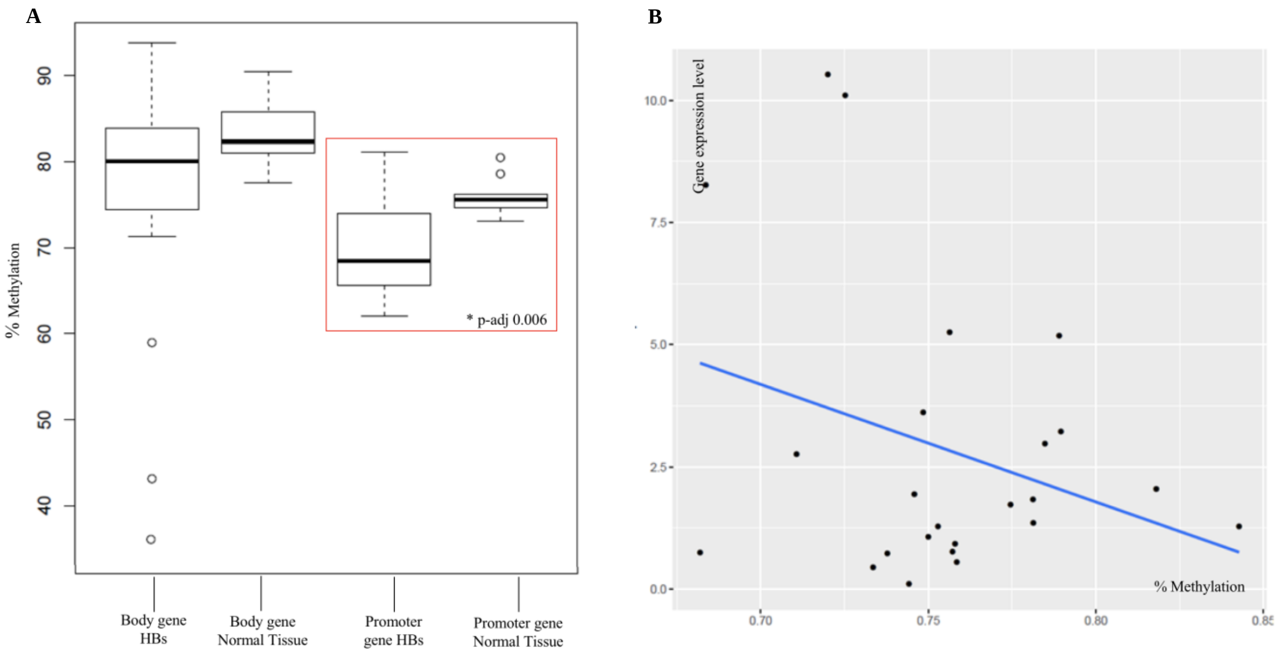

Supplementary Figure 6: ***CX3CL1*** **DNA methylation data recovered for the same set of HB samples from our previous work (38) and correlation with gene expression data: a.** A significant DNAm difference was observed in *CX3CL1* gene promoters of HBs in relation to control livers (p-adj 0.006). **b.** An inverse correlation between gene expression and DNAm level was detected in *CX3CL1* gene body (Spearmans rho 0.46, p-value 0.02).

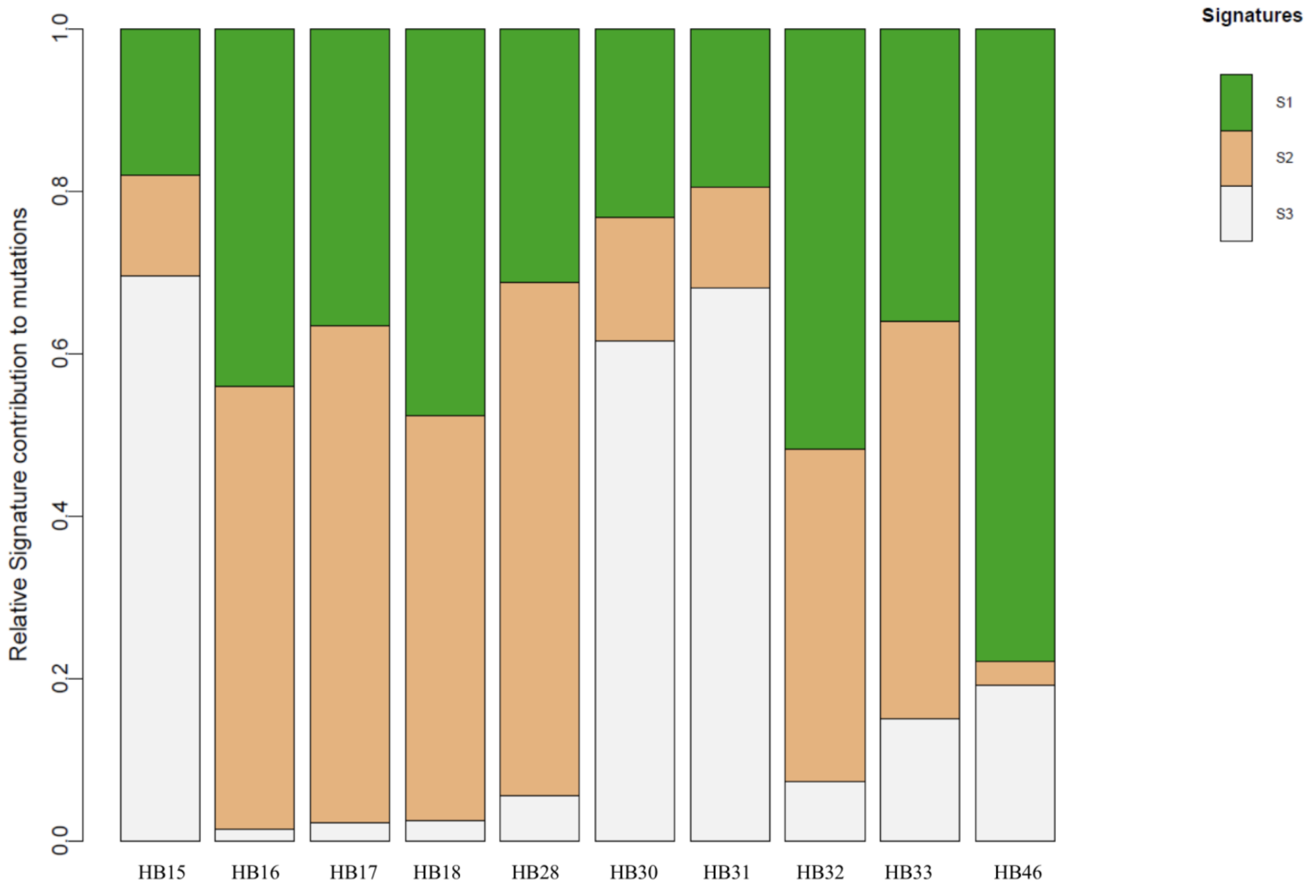

Supplementary Figure 7: Relative signature contribution to mutational profile of each hepatoblastoma sample.

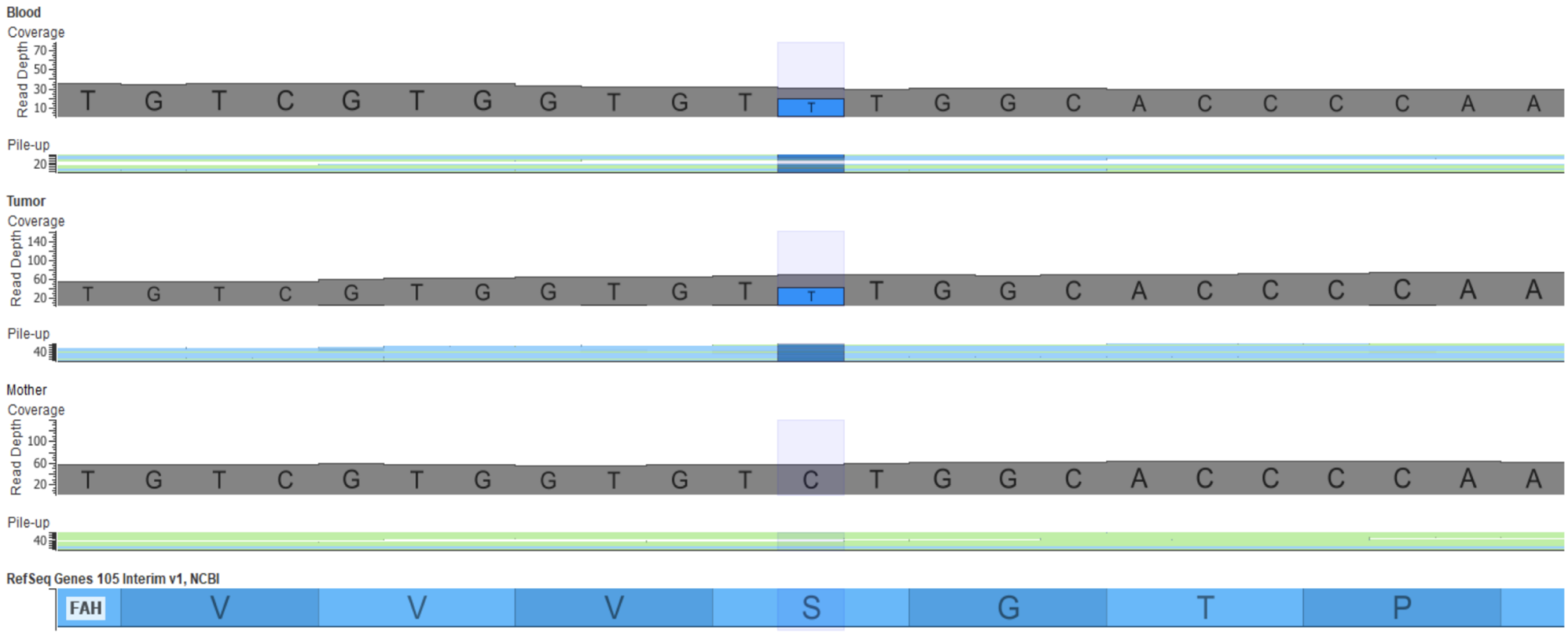

Supplementary Figure 8: Germline *FAH* likely pathogenic variant detected by exome sequencing in the Patient HB33 (congenital hepatoblastoma). BAM file images showing a germline C>T variant (blue) in the exon 6 of the *FAH gene*, present in heterozygosity in the patient's blood and tumor, and absent from the clinically normal mother. This missense variant (p.Ser169Phe) is predicted as pathogenic by 6 out of 6 *in silico* tools, and was not previously reported in any consulted population databases.

A

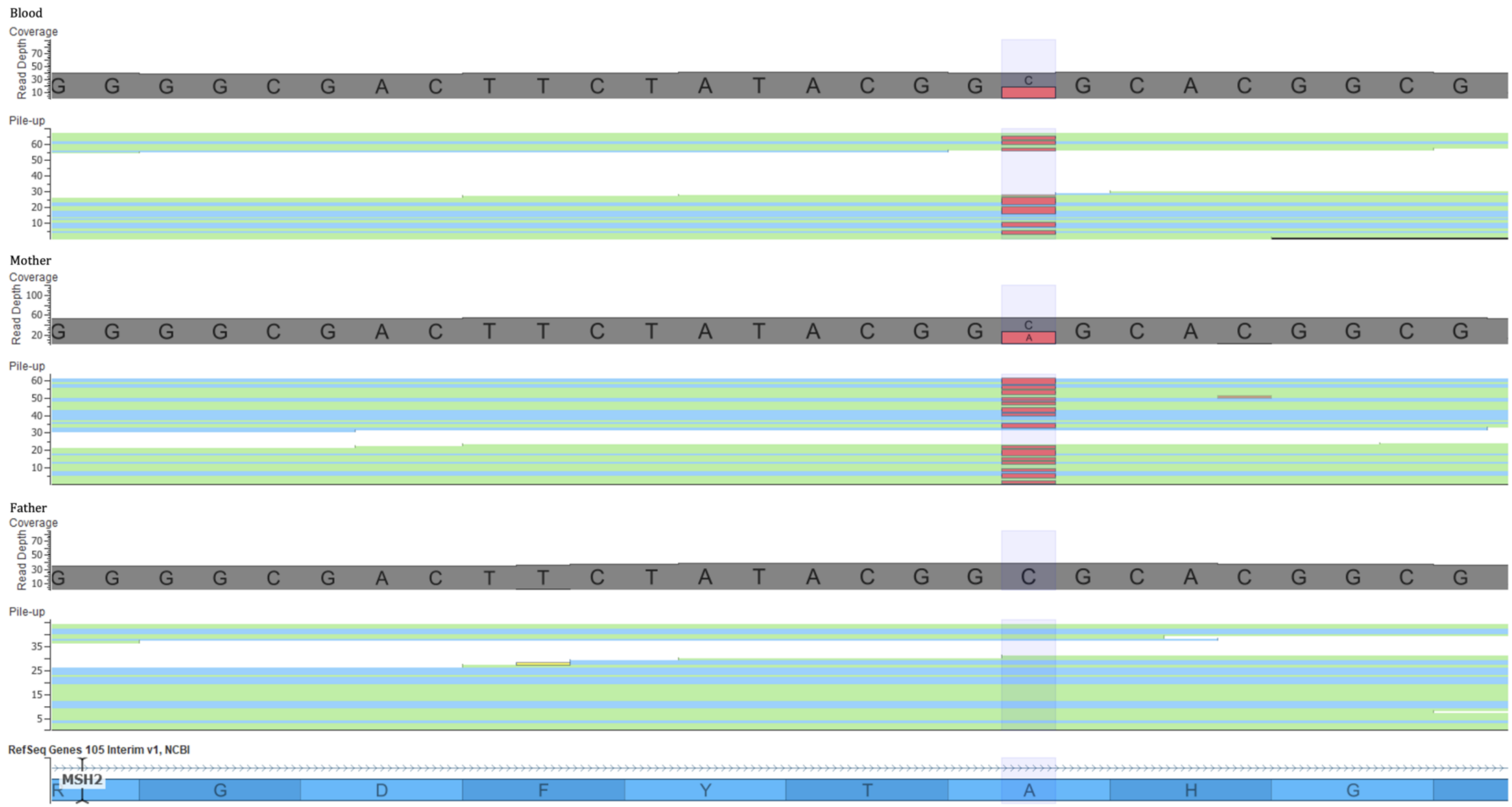
Blood
Coverage
Read Depth
Pile-up
Mother
Coverage
Read Depth
Pile-up
Father
Coverage
Read Depth
Pile-up
RefSeq Genes 105 Interim v1, NCBI
MSH2
G G G G C G A C T T C T A T A C G G C G C A C G G C G
G D F Y T A H G

B

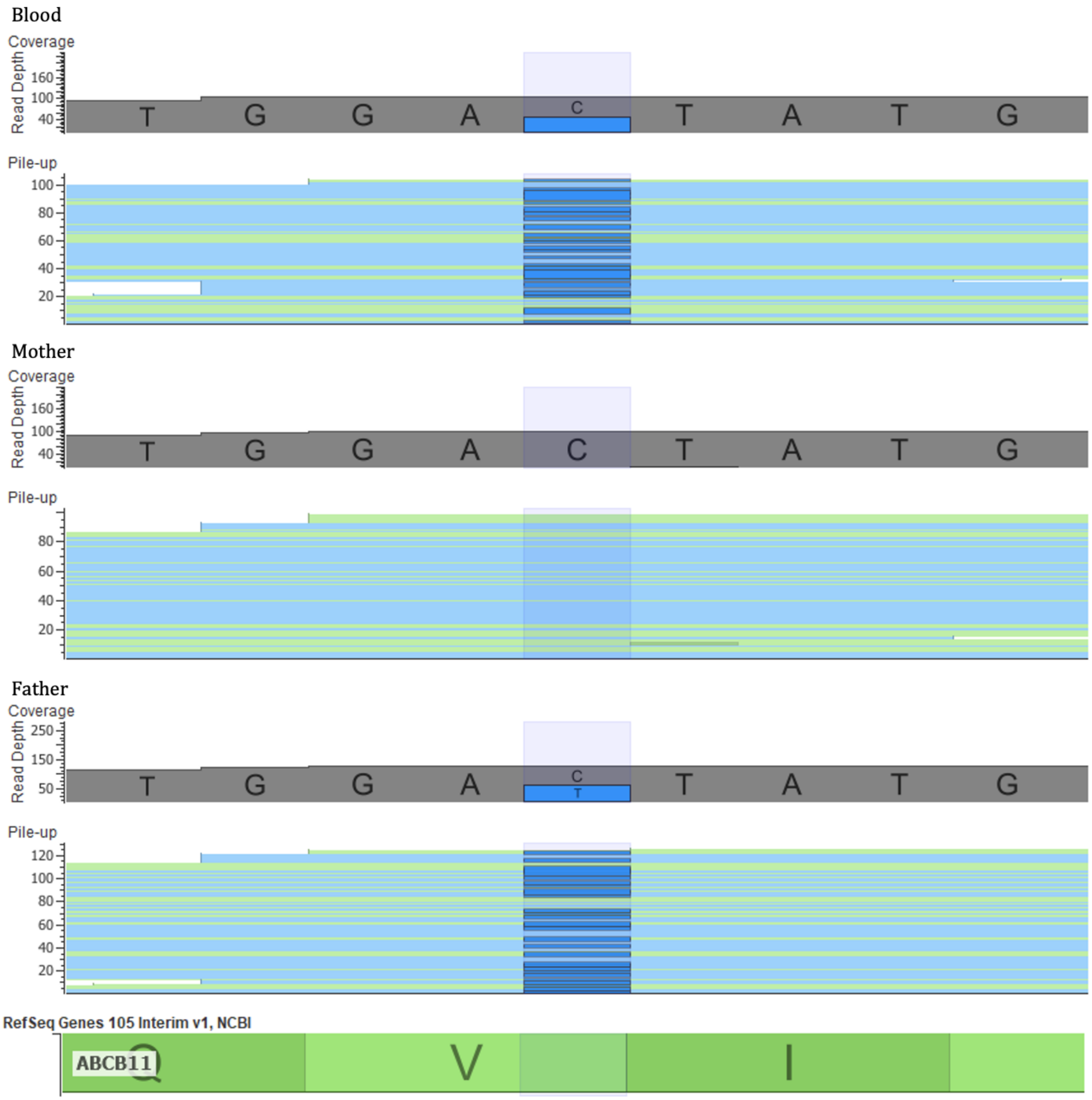
Blood
Coverage
Read Depth
160
100
40
T G G A C T A T G
Pile-up
100
80
60
40
20
Mother
Coverage
Read Depth
160
100
40
T G G A C T A T G
Pile-up
80
60
40
20
Father
Coverage
Read Depth
250
150
50
T G G A C T T A T G
Pile-up
120
100
80
60
40
20
RefSeq Genes 105 Interim v1, NCBI
ABCB11
Q
V
I

Supplementary Figure 9: Germline rare variants detected by exome sequencing in the Patient HB46 (syndromic patient).

- **(A)** BAM file images showing a germline C>A variant (red) in the exon 1 of the *MSH2* gene, detected in heterozygosity in the patient's blood and inherited from his mother. The missense variant (p. Ala45Glu) is predicted as pathogenic by 6 out of 6 *in silico* tools and was not previously reported in any consulted population databases.
- **(B)** BAM file images showing a germline C>T variant (blue) in the exon 14 of the *ABCB11* gene, detected in heterozygosity in the patient's blood and inherited from his father. The missense variant (p. Val529Ile) is predicted as pathogenic by one *in silico* tool and is very rare in consulted population databases.

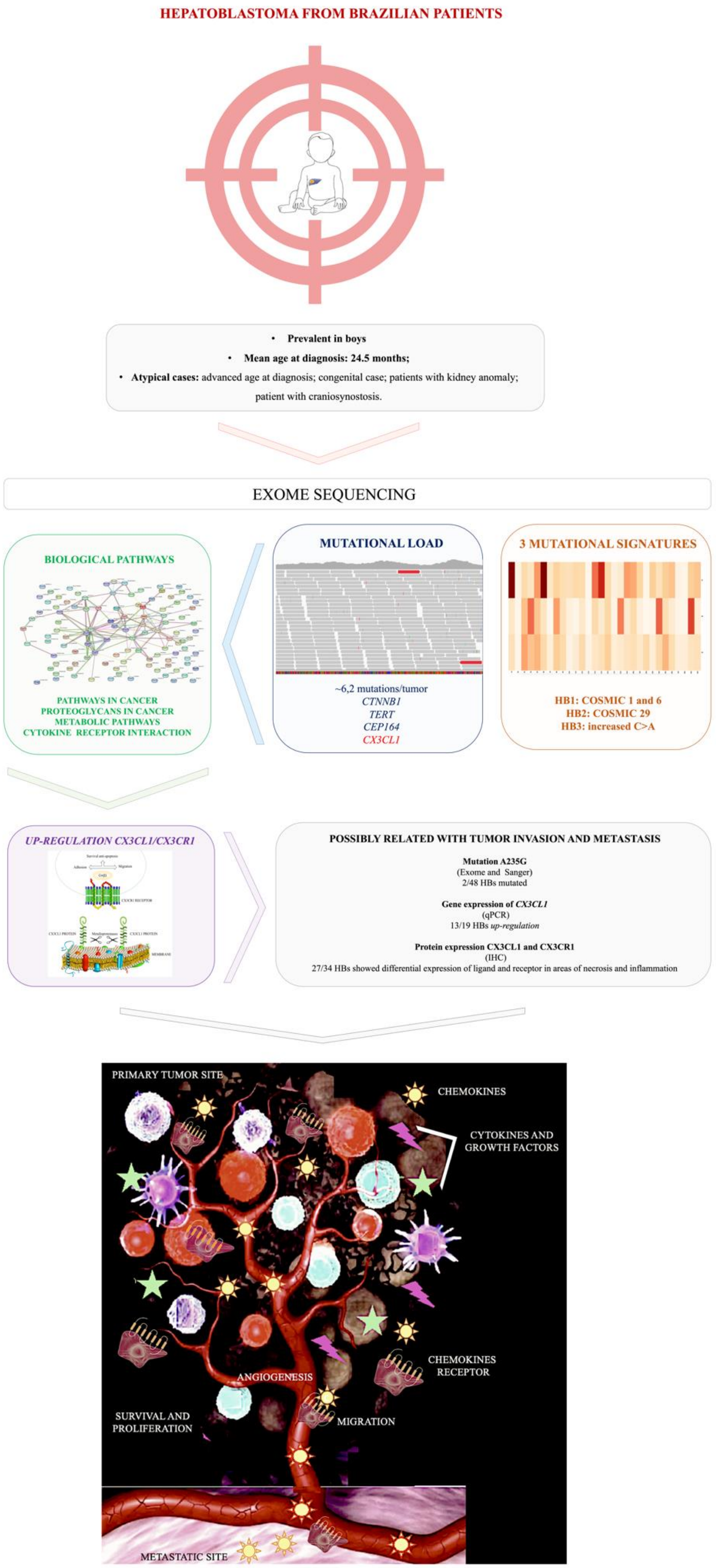

Supplementary Figure 10: Graphical abstract summarizing the findings.
